# Palliative care research on the island of Ireland over the last decade: a systematic review and thematic analysis of peer reviewed publications

**DOI:** 10.1186/1472-684X-12-33

**Published:** 2013-09-04

**Authors:** Sonja J McIlfatrick, Tara Murphy

**Affiliations:** 1 All Ireland Institute of Hospice and Palliative Care c/o Education and Research Centre, Our, Lady’s Hospice and Care Services, Harold’s Cross, Dublin 6w, Ireland; 2 Institute of Nursing Research/All Ireland Institute of Hospice & Palliative Care, University of Ulster, Room 12 J11 Shore Road Newtownabbey Co., BT37 OQB, Antrim, Northern Ireland

**Keywords:** Systematic review, Palliative care, Hospice care, End-of-life, Research

## Abstract

**Background:**

As palliative care research continues to expand across Europe, and the world, questions exist about the nature and type of research undertaken in addition to the research priorities for the future. This systematic review, which is the first stage of a larger scale study to identify the research priorities for palliative care on the island of Ireland, examined palliative care research conducted on the island over the last decade.

**Methods:**

A comprehensive search strategy was implemented and strict eligibility criteria were applied in order to identify relevant peer-reviewed journal articles. Inclusion criteria were all of the palliative care studies undertaken on the island of Ireland and published between January 2002 and May 2012. These were assessed in relation to year, setting, sample size, research methodology, and relevant findings.

**Results:**

412 publications were identified for screening and their abstracts obtained. After eliminating articles that did not meet the inclusion criteria, 151 remained for further analysis. A thematic analysis of 128 studies published between 2006 and 2012 revealed eight core themes: (1) specific groups/populations; (2) services and settings; (3) management of symptoms (physical, psychological, social); (4) bereavement; (5) communication and education; (6) death and dying; (7) spirituality; and (8) complementary and alternative medicine/intervention (CAM). There was an upward trend in the number of publications in palliative care research over the last ten years with over 72% of studies being published within the previous four years. A slightly higher number of studies were quantitative in nature (surveys, questionnaires, standardised assessments) followed by qualitative (individual and focus group interviews, case studies, documentary analysis and retrospective case note reviews), mixed methods, and systematic reviews.

**Conclusions:**

Whilst there has been a welcome growth in palliative care research across Ireland, this has largely been needs-based and small scale studies. In contrast, international researchers and decision makers recommend the need for more outcomes focused multidisciplinary research. An examination of palliative care research is an essential first step in seeking to develop future priority areas for further research, highlighting opportunities for future collaboration both nationally and internationally.

## Background

Changing demographic trends including an ageing population, increased life expectancy for individuals living with life-limiting illnesses, and the impact of illness on individuals’ physical, psychological and social wellbeing means that the demand for high quality palliative care services is expected to increase [1]. Estimates from Europe indicate that 3.2 million European citizens are newly diagnosed with cancer and that 1.7 million deaths occur annually [2]. The World Health Organisation (WHO) (2003) predicted that the number of people dying from cancer each year will increase to over 2.1 million by 2020 (a 24% increase) [3]. A similar pattern may be noted nationally. For example, in the United Kingdom, a 17% increase in the annual number of deaths is expected, along with an increase in the percentage of deaths of those aged 85 years or older, from 32% in 2003 to 44% in 2030 [4]. Data from the World Cancer Research Fund International indicates that cancer cases in Ireland could increase by as much as 72% by 2030 [5]. According to Ireland’s Central Statistics Office [6], the Irish population is gradually ageing with a predicted doubling of numbers for those aged 85 years by 2021 [6]. This is predicted to coincide with an increase in disease prevalence between 2007 and 2021 for diseases including cancer as well as chronic conditions such as cardiovascular and respiratory diseases [7]. Data such as this has serious implications on palliative care service provision as estimates show that each year, 1.6 million cancer patients in Europe experience pain related to their disease, one third of them will require complex treatment and a significant number also experience distressing symptoms [8]. This picture is further complicated by ongoing economic challenges, resulting in cuts to services, staff numbers and resources. Such rising health care costs make it even more critical for services to demonstrate their effectiveness [9,10]. Research that can inform evidence-based practice and demonstrate improved outcomes and cost effectiveness for a range of services and interventions in palliative care is therefore required [9-11].

Palliative care is defined by the WHO as an approach that improves the quality of life of patients and their families facing the problem associated with life-threatening illness through the prevention and relief of suffering by means of early identification and impeccable assessment and treatment of pain and other physical, psychosocial and spiritual problems [12]. Within Europe it has been noted that variations persist in terms of access to care, care quality, and research infrastructure. The quality-of-death index (2010) that ranks countries according to their provision of end-of-life care, classified the United Kingdom and Ireland first and fourth respectively [13]. Whilst this may be beneficial, questions exist around the nature and type of research being undertaken. Higginson [10] argued that palliative care would benefit substantially from more outcome-based research, using a range of methods, for a diverse range of illnesses and populations. However Chalmers & Glasziou [14] raise concerns about the extent to which health-focused outcomes as prioritised by researchers would be considered priorities by patients, families or clinicians.

Undeniably, the consensus among international research experts, groups and lead organisations in palliative care is that there is a pressing need for clinically-relevant, collaborative, interdisciplinary, and strategic approaches to palliative care research [10,15-20]. For example, the National Hospice and Palliative Care Organisation (NHPCO) [19] based in the United States, emphasised the need for collaborative research activities but equally recognised the challenges that can occur when the academic and clinical worlds collide. Both Higginson [10] and Kaasa [21] emphasised the need for collaborative research at all levels as well as considerations for research capacity-building.

It is evident that research in palliative and end-of-life care at all levels and in particular outcomes-focused and cost effectiveness research is needed in order to improve service delivery and ultimately, optimise patients’ quality of life. With a growing ageing population, an increase in the prevalence of chronic diseases, and the consequences of both in terms of medical expenditure, it is anticipated that the need for palliative and end-of-life care will intensify resulting in a greater need for research in this area. Within the context of considering research priorities in palliative care and the establishment of a new All Ireland Institute of Hospice and Palliative Care (AIIHPC) (http://www.aiihpc.org), the authors set out to explore the nature of palliative care research conducted on the island of Ireland. This was undertaken in order to examine the relevance and overall contribution of the research as well as to identify future research priorities for palliative care in Ireland.

### Aims

The aim of the systematic review was to identify peer-reviewed journal articles that described palliative care research studies undertaken on the island of Ireland over the last decade. The review was led by AIIHPC which seeks to improve palliative care experience on the island of Ireland, hence the rationale for focusing on studies undertaken in Ireland. The specific objectives of the review were:

a) To source the literature published between January 2002 and May 2012.

b) To provide a thematic analysis of peer-reviewed journal articles published over the last five years.

c) To examine study characteristics in terms of setting, sample, research methodology, and relevant findings.

d) To contextualise palliative care research on the island of the Ireland internationally.

## Methods

### Search strategy

The review was undertaken to identify research conducted in palliative care across the island of Ireland, within the last 10 years. The following standard bibliographic databases were searched (CINAHL, MEDLINE, SocINDEX, PsychInfo, PUBMED, Cochrane and ScienceDirect as well as the Irish Health Repository ‘LENUS’). A combination of the following search terms were used with the Boolean phrase ‘and/or’ in order to maximise the type and range of material captured in the search: (“palliative” or “hospice” or “terminal” or “end-of-life”), (“palliative care” or “hospice care” or “terminal care” or “end-of-life care”), (“dying” or “death” or “terminal” or “terminally”), (“palliative” or “palliation” or “palliating” or “palliate” or “palliatively” or “palliat”), (“malignant” or “non-malignant”), (“pain” or “pain management” or “pain prevention” or “pain control”), (“symptom(s)” or “symptom management”), (“bereavement” or “bereaved” or “grief” or “grieving”), (“service design” or “service delivery” or “intervention”). Additional search terms ‘Ireland’, ‘Northern Ireland’ or ‘Irish’ were applied in order to increase the sensitivity of the search. The bracketed groups were combined in multiple ways in order to capture the widest range of studies relevant to palliative care. For example, (“palliative” or “hospice” or “terminal” or “end-of-life”) combined with (‘Ireland’, ‘Northern Ireland’ or ‘Irish’).

Publications were selected based on the following inclusion criteria:

a) Research output published between January 2002 and May 2012;

b) Research conducted on the island of Ireland;

c) Research relevant to palliative care.

A general review of key hospice and palliative care websites in Ireland and Northern Ireland was also conducted. In two cases, information managers/librarians were contacted directly about research funded and/or published by their organisations within the last decade. Given the volume of documents initially generated, the search was restricted to full text articles and systematic reviews published in peer reviewed journals thereby excluding other types of publications such as conference papers, newsletters, editorials, commentaries, discussion documents, and unpublished theses. Given that the focus of the current paper was on research conducted on the island of Ireland in which the first language is English, studies had to be published in English. The titles and abstracts of publications returned in the database results were manually reviewed in order to confirm their relevancy to palliative care; that they were research-focused; and were studies conducted on the island of Ireland.

### Data extraction

All citations generated through the search were exported to Endnote©. Full text articles were retrieved where available in order to facilitate the review process. Citations were initially organised according to date (i.e. pre and post 2006) and reference type (i.e. journal article, conference paper, report, book, newsletter, editorial, etc.). All journal articles were formally included in the review. Duplicates were removed where there was more than one article reporting on the same study since the reviewers wanted to avoid skewing the review by including multiple papers reporting on the same or similar findings. In this case, the earliest paper was selected and subsequent papers reporting on the same findings were excluded from the review. An initial review of the articles separated them into seven broad thematic headings that best described the research focus: (1) understanding palliative care; (2) treatment and intervention; (3) training and education; (4) service delivery; (5) measurement and evaluation; (6) methodological and ethical issues; and (7) strategy and policy. The reviewers, together and separately, categorised the articles under one of these headings with a view to refining the headings further into the process.

The authors used the papers retrieved to generate and set the themes and subthemes identified within this review. Recognising that some papers may have multiple domains, papers were categorised based on the primary aims and outcomes of studies. In the case of a more complex paper such as one that aims to compare the needs of heart failure and cancer patients, the study’s outcomes were examined in more detail in order to determine whether the primary objective of the researcher was to contextualise the findings within a heart failure or cancer population. In this case, the authors found that the primary focus of the paper was on heart failure and consequently the paper came under the theme ‘specific groups’ and subtheme ‘heart failure’. Where agreement was not reached, the reviewers examined the full text article where available and re-categorised.

A second review of the articles identified additional studies that were deemed not to meet the inclusion criteria. For example, studies drawing on Irish subsamples; reporting on service evaluation and audit as part of an education/curriculum development initiative; outlining study protocols; as well as literature reviews. The current paper prioritised studies led by researchers on the island of Ireland since the purpose of the review was to establish a baseline of research in palliative care and identify any gaps. Studies that included Irish subsamples were international and tended to synthesise the findings in order to report on outcomes at a continental or global level. In terms of staff feedback surveys, the majority of these were based on very small sample sizes and the findings applicable to only that setting or service. Therefore, these were excluded on that basis.

A list of the remaining studies was developed and data were gathered on setting and/or sample size, research methodology (design/methods), standardised instruments (where applicable), and relevant findings. The third and final review of articles involving re-examination of the original thematic headings identified eight core and more meaningful categories which are reported below.

## Results

**Figure 1:**
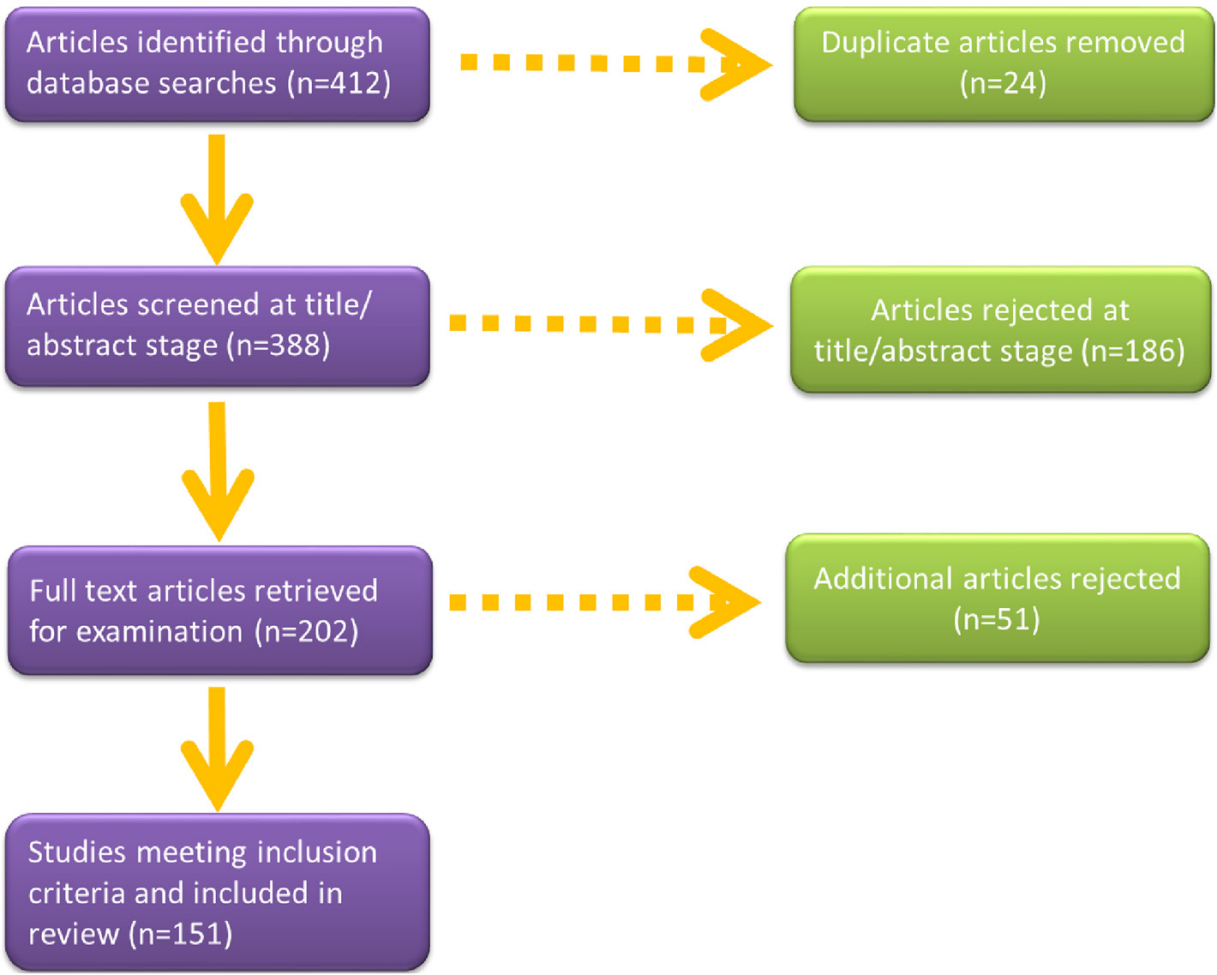
Selection and review process.

A flow diagram detailing the selection of studies is detailed in Figure 1. The electronic literature searches identified 412 potential publications for screening after which duplicates were removed. The titles and abstracts of the remaining 388 publications were reviewed as well as publication type. This resulted in a total of 186 documents being excluded from the main review including books/book sections, conference papers/abstracts, newspaper articles/bulletins, and discussions/editorials/commentaries/journal articles (e.g. literature reviews; study protocols; discussions). Following this, 202 peer-review journal articles were identified for screening. A second review of abstracts for relevancy found that 51 articles did not meet the inclusion criteria. The final review included 151 studies published over the period under examination.

**Figure 2:**
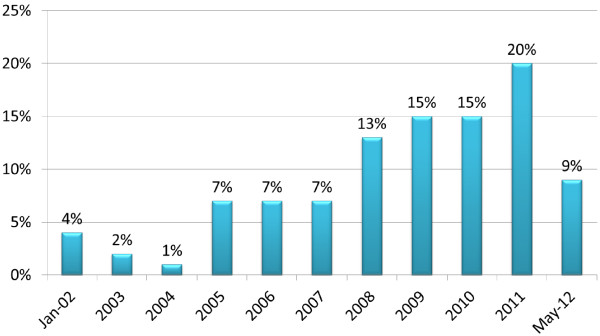
Percentage of peer-reviewed articles by year of publication.

**Figure 3:**
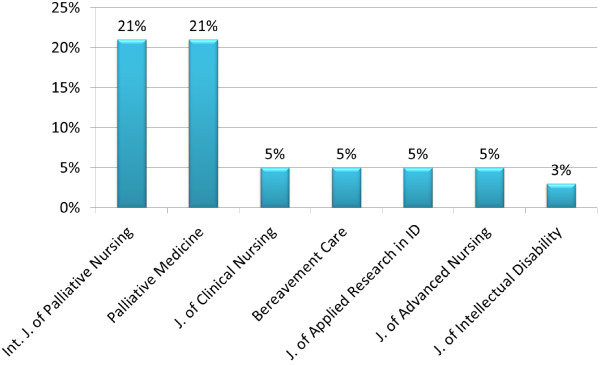
Top 7 most popular journals (2002–2012).

There was an upward trend in the number of studies in palliative care research published from January 2002 to May 2012 with over 72% being published within the previous four years (Figure 2). Papers were published in a total of 86 national and international journals the most popular being the International Journal of Palliative Nursing (21%) and Palliative Medicine (21%) (Figure 3). Interestingly, there was an absence of studies on palliative care in Palliative Medicine pre-2006 relating to the island of Ireland. Other popular journals included the Journal of Clinical Nursing (5%), the Journal of Advanced Nursing (5%), and two journals in the area of intellectual disability research i.e. Journal of Applied Research in Intellectual Disability (5%) and Journal of Intellectual Disability Research (3%).

**Figure 4:**
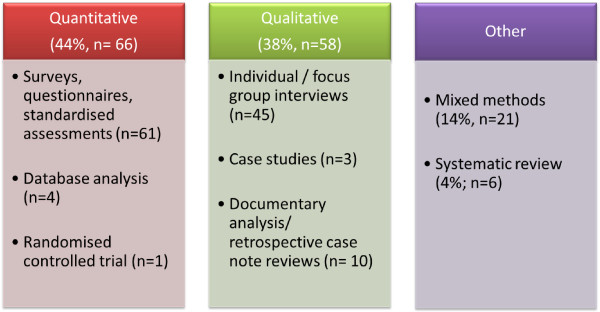
Research methods in studies published between 2002 to 2012.

In terms of research design, 44% of studies were quantitative in nature compared to 38% that were qualitative (Figure 4). Other study designs included mixed methods (14%) and systematic reviews (4%). Surveys and questionnaires were the primary quantitative research methods employed while the most frequently used qualitative methods were individual and focus group interviews. For study characteristics see Additional file 1: Table S1 and Additional file 2: Table S2.

There was wide variation in terms of sample size across the individual studies with the majority based on samples sizes of less than 100. Studies were undertaken in a range of settings including acute hospitals, the community/home, generalist and specialist services (e.g. intellectual disability, cancer, and palliative care), hospices, and nursing homes.

For the purposes of the current review, the authors focused the thematic analysis on studies published between January 2006 and May 2012. This was undertaken for pragmatic reasons as well as coinciding with a time frame of significant clinical and policy development for palliative care practice. Eight core themes emerged after re-examination of the remaining articles (n = 128): (1) specific groups/populations; (2) services and settings; (3) symptoms (physical, psychosocial); (4) bereavement; (5) communication and education; (6) death and dying; (7) spirituality; and (8) complementary and alternative medicine/intervention (CAM) (Table 1).

**Table 1:** Area of palliative care research breakdown according to publications (2006–2012)

**Core themes (N = 128)**	**Subthemes (N = 128)**	**Examples**
**Specific groups (n = 51)**		
	Children (n = 11)	Staff experiences; bereavement; symptoms; needs; service delivery
	Intellectual disability (n = 10)	Bereavement; staff training; carers’ perceptions & experiences; dementia
	Malignant disease (n = 7)	Palliative management; quality of life
	COPD (n = 6)	Education programme; needs and experiences; carers
	Dementia (n = 3)	Health economics; self-efficacy
	Heart failure (n = 3)	Nutrition; needs and experiences
	Older people (n = 3)	Staff needs; attitudes; service models
	Parkinson’s disease (n = 4)	Carers’ experiences
	Non-malignant disease (n = 2)	Needs; service barriers
	Same-sex couples (n = 1)	Bereavement
	Travellers (n = 1)	Experiences
**Management of symptoms (n = 27)**		
	Delirium (n = 13)	Profiling; predictions; depression; assessment tools
	Pain (n = 7)	Chronic pain; attitudes & barriers to pain management; help-seeking behaviour; cost
	Cachexia (n = 2)	cancer care; needs; nutrition
	Nutrition (n = 2)	Survival; weight assessment
	Psychological (n = 2)	Use of CBT techniques; stress
	Other physical (n = 1)	Symptom reporting
**Services &settings (n = 27)**	QoL and acute hospitals care; multi-visit service; palliative care unit; roles and responsibilities; waiting times; medication	
**Bereavement (n = 10)**	Needs assessment; bereaved partners; workplace; support	
**Communication &education (n = 4)**	Information/education needs; professional development; blogging	
**Death &dying (n = 5)**	Public awareness; moment of death and experiences;	
**Spirituality (n = 3)**	Staff experiences; chaplaincy service; religion	
**CAM (n = 1)**	Oncology	

Research focusing on specific groups (40%, n = 51) was the most popular theme followed by symptoms (21%, n = 27), and services and settings (21%, n = 27). Specific groups or subthemes such as children (11 studies), intellectual disability (10 studies), and malignant disease (seven studies) were most popularly researched populations while there were noticeably fewer studies on minority groups such as same sex couples (one study), individuals from the Travelling Community (one study), and those with non-malignant illnesses (two studies). A common thread underlining this theme was the exploration of the needs, experiences and perceptions of service users, carers and professionals in relation to illness and palliative care services. Similarly, there were several studies examining bereavement in relation to children and separately, intellectual disability populations. While the reviewers in this review identified ‘bereavement’ as a separate theme in itself, the latter had a more generic focus.

‘Management of Symptoms’, ranging from physical to psychosocial manifestations, was the focus of 27 studies. The most frequently researched areas were delirium (13 studies) and pain (seven studies), followed by cachexia (four studies), nutrition (two studies) and psychological symptoms (two studies). Interestingly studies assigned to this theme were more likely to implement standardised instruments in the gathering of data than any other themes. Research into services and settings (21%, n = 27 studies) addressed issues such as quality of life, roles and responsibilities of professionals, and delivering palliative care in general and specialist settings. Other notable themes of research included bereavement (8%, 10 studies), death & dying (4%, five studies), communication & education (3%, four studies), and spirituality (2%, three studies). Only one study included in the current review focused on CAM.

## Discussion

The findings from this study clearly show that palliative care research is on the increase in Ireland particularly over the last five years. This is also reflective of the international perspective. Various reasons have been suggested for the universal increase in palliative care research, such as the recognition of palliative care and its contribution as evidenced by WHO [12]; investments in some well-funded palliative care research initiatives in Europe, Canada, the USA and Australia [22-26] and global developments in mapping levels of palliative care development [27]. According to Sigurdardottir et al. [20] the state of palliative care research can be closely linked to the development of clinical palliative care services in a country or a region. They argued that there is a strong link between specialisation, training and research and that recruitment is a significant issue. Therefore establishing palliative medicine as a specialty as well as other relevant professional staff is essential for both improving capacity not only for clinical work but also for research. There is little doubt that such developments in Ireland, as evidenced within the Eurobarometer ratings for palliative care (Ireland ranked 5th) [28] and the Quality of Death Index (ranked 4th) have contributed to the increase in palliative care research undertaken in Ireland over the last five years. There are, however, questions as to whether the developments in palliative care research are in line with these ratings for palliative care provision.

The findings also clearly indicate a diverse range of research activity. This ranges from death and dying, symptom assessment, bereavement, communication to complementary and alternative approaches and a focus on different groups and populations. This diversity would be indicative of the different disciplines involved in palliative care research such as psychology, psychiatry, palliative medicine, nursing, general practice, social science and health services research, amongst many others. Whilst initially such diversity could be considered a weakness it can be equally considered as strength. For it is only through diversity, with a mixture of competences and perspectives, that complex research questions within the area of palliative care can be addressed both nationally and internationally.

### Specific groups and settings

The results demonstrate that major areas of research undertaken were focused on specific groups and populations as well as different settings. These groups included children, intellectual disability and non-malignant illnesses. The latter group can be seen to address the need to move beyond cancer and undertake palliative care research in relation to chronic diseases such as heart failure, coronary obstructive pulmonary disease (COPD), renal and liver diseases and dementia. Such a need was identified by Higginson [10] in an examination of palliative care services and international research agendas. This does reflect a changing paradigm for palliative care, where it is increasingly recognised that palliative care is appropriate and potentially beneficial at the time of diagnosis and for other conditions. Furthermore, this could be indicative of changes in demography. Interestingly, there were fewer studies in settings involving primary care despite the growing preference for patients to be cared for at home.

### Management of symptoms

A recent pan European survey of research in end of life cancer care, across 41 European countries (n = 127 responses), found that the most frequently reported active research areas were pain, assessment and measurement tools, last days of life, and quality of death [20]. This focus on symptomatology (specifically pain, cachexia, fatigue, cognitive symptoms and delirium, breathlessness) was previously identified as one of the main areas for future research in a workshop held by PRISMA (priorities for research and measurement in end of life care). This workshop involved 30 research experts in palliative care/medicine (85% from a medical background) representing 25 European countries [20]. According to Kaasa et al. [29] there is a need for research on the aetiology of symptoms, the development and testing of standardised instruments, and the development of evidence-based guidelines to inform clinical practice. This is in line with a European Parliamentary report which advocated for palliative care research that focused on the aetiology and management of symptoms and holistic approaches to palliative care [30]. Research focusing on symptom management was the second largest theme for palliative care research undertaken in Ireland with the two most researched domains being delirium and pain. It is noteworthy that symptom management was primarily focused on domains in which there are key champions and leaders on the island. The continued research focus on this theme is essential for palliative care practice with a clear need to undertake clinically relevant research, clearly aligning clinical and research agendas. Furthermore, there appears to be an absence of user/carer involvement in palliative care research in Ireland which is significant given the requirements for patient/public involvement by funders and those commissioning research.

### Health services research

The third key area of published research undertaken in Ireland was focused around service provision and health settings. Health services research that informs health care policy, planning and provision was also identified as a key area for future research in the PRISMA workshop [9]. It is interesting to note that a UK focused consultation for palliative care research demonstrated a greater emphasis on health economic aspects and service delivery [31]. A rationale for this may be the greater involvement of service funders and providers in the consultation. Regardless however, of the reasons behind this focus, the importance of the health economic aspects of palliative care research cannot be underestimated. This is of great significance within the context of rising health care costs and the need for services to demonstrate their effectiveness [9]. Recent findings arising from an international Think Tank on global research priorities in health economics for palliative care identified four priority areas including (1) models and components of care and setting (e.g. rural vs. urban); (2) evaluation studies; (3) methodological considerations (e.g. analysing and interpreting economic data, development of agreed terminology); and (4) strategies for initiating studies and use of data [32].

### Nature of research

The findings from this study demonstrate some clear issues in terms of methodological rigour, a lack of measurement of clear outcomes and an over emphasis on needs based research. It was found that the studies were largely descriptive in design, (including both quantitative and qualitative research), with a wide variation in sample size and a lack of use of recognised standard measures and consideration of key outcomes. This indicates a paradox. Whilst it is acknowledged that research in palliative care is complex with difficulties in measuring multiple symptoms alongside ethical concerns, there is a need to focus on outcomes based research. Such outcomes measures need to be patient centred, valid, reliable and applicable for both complex and more ‘simple’ intervention studies [21]. However, many outcomes measures such as quality of life or satisfaction are considered highly subjective and influenced by a range of variables including socioeconomic background, culture, gender, and so on [10]. Kaasa et al. [17] and later Kaasa et al. [21] highlighted the significant role that systematic reviews and randomised controlled trial (RCT) studies have in providing reliable scientific evidence and enabling researchers/clinicians to compare data and undertake meta-analyses. This appeared to be an area of deficit and one that requires further development for Ireland in terms of research capacity building. An increase in methodological rigour, with a focus on RCTs and systematic reviews is necessary in order to establish the evidence base for best practice.

### Limitations

One of the limitations of the systematic review is that the key words used to select publications may not have captured other studies in the area of palliative care which used different terminology. This may have excluded relevant peer-reviewed papers due to them not mentioning a keyword in their title, abstract or subject terms. A further limitation is that while this review aimed to identify trend patterns for palliative care research, the exclusion of non-peer reviewed publications including commissioned reports may have resulted in the over or under representation of certain themes or subthemes.

## Conclusions

This study provided baseline evidence of the nature and type of palliative care research undertaken within Ireland over the last decade. Some areas of significant strength were noted such as a clear focus to address the needs of specific groups and populations, focusing on palliative care beyond cancer. Furthermore, there was also evidence of research focusing on symptom management and palliative care service developments. Despite this, however, the current review identified several research gaps specific to palliative care including public health and policy research. While a number of papers reported on methodological and ethics issues, these were not included as they were literature reviews or reflective pieces. There was also a lack of multidisciplinary and outcomes-focused research in this area which may reflect both the relative currency of palliative care research in comparison to other countries such as the UK, US and Canada.

The research undertaken was clearly limited by issues relating to methodological rigour with a lack of measurement of outcomes, overemphasis on needs based research and limitations associated with sampling. Similar issues have been identified at a European and global level. Such an analysis is useful when seeking to identify areas for future research and research priorities. There is a clear need for future strategic direction and collaboration for palliative care research both nationally and internationally. Collaboration between palliative care researchers in the island of Ireland remains in its infancy. There is a need to maximise existing resources, leverage expertise and capability, and build critical mass in order to produce excellent, internationally-competitive research.

## Competing interest

The authors declare that they have no competing interest.

## Authors’ contributions

SMcI made a considerable contribution to the conception and design of the study, analysis, interpretation, revisions and final approval of the manuscript. TM made a considerable contribution to data collection, analysis, interpretation, drafting and final approval of the manuscript. All authors read and approved the final manuscript.

## Pre-publication history

The pre-publication history for this paper can be accessed here:

http://www.biomedcentral.com/1472-684X/12/33/prepub

## Supplementary Material

Additional file 1: Table S1 Published palliative care research on the island of Ireland (2002–2005). Click here for file

Additional file 2: Table S2 Published palliative care research on the island of Ireland (2006–2012). Click here for file

## Statement of adherence to the MOOSE guidelines

The authors of this paper state that they have adhered to the MOOSE guidelines (2000) for systematic reviews.
