# Supplementary material for: Palliative care research on the island of Ireland over the last decade: a systematic review and thematic analysis of peer reviewed publications
Source: BMC Palliat Care. 2013 Sep 4;12:33. doi: 10.1186/1472-684X-12-33 (PMC3848123; doi:10.1186/1472-684X-12-33)
Supplement: Additional file 1: Table S1 — Published palliative care research on the island of Ireland (2002–2005). [file 1472-684X-12-33-S1.doc]

**Table 1. Published palliative care research on the island of Ireland (2002-2005)**

| **Author, Year** | **Aim** | **Sample/ Setting** | **Research Design** | **Method** | **Outcome measures** | **Key Findings of the Study** |
| --- | --- | --- | --- | --- | --- | --- |
| **SPECIFIC GROUPS (NON-MALIGNANT)** | | | | | | |
| **Cleary, J. et al (2005)** | (a) To measure the overall quality of life of people receiving haemodialysis, (b) To compare the quality of life of the sample with that of the general population & (c) to identify any differences between the quality of life of people who are adequately dialysed and those inadequately dialysed. | Patients (n=97) undergoing haemodialysis treatment at a hospital | Quantitative | Survey | 36-item Short Form Health Survey | Patients receiving haemodialysis identified limitations in a number of areas including vitality, physical functioning and physical role limitations. They also reported significantly lower physical functioning when compared with general population norm-based scores. Differences were also found in mental health scores between patients who were well-dialysed and those less well-dialysed. |
| **SPECIFIC GROUPS (MALIGNANT)** | | | | | | |
| **Kealey, P. et al. (2005)** | An evaluation of the domiciliary occupational therapy service provided for patients in the palliative stage of cancer care in North and West Belfast from the perspective of the patients and carers. | Patients (n=30) & carers (n=30) | Quantitative | Survey | Easi-Quest Patient and Staff Surveys, Reference  Point Ltd), occupational therapy outcome measures | High levels of satisfaction reported, however, there are gaps identified in service provision and a lack of clear information among patients and their carers about the role of the occupational therapist and the range of services they can provide. |
| **Kavanagh, E. et al. (2004)** | To describe case of a patient with a primary colonic adenocarcinoma who developed calcified pulmonary metastases while receiving palliative chemotherapy. | Patient (n=1) | Qualitative | Retrospective clinical case note review | n/a | Calcified pulmonary metastases are described in association with a variety of primary neoplasms. |
| **O’Connor, A. et al. (2004)** | To report on the successful use of expandable metal stents in two cases of malignant gastric outlet obstruction. | Two patients | Qualitative | Case studies | n/a | The use of endoscopic stents as a primary palliative procedure has been well documented. Two cases are presented here, which highlight the benefits of this procedure in patients who develop recurrence at the site of a previous palliative gastroenterostomy. |
| **Curt, G. et al. (2003)** | To assess cancer-related fatigue and compare to international findings | 109 physicians &  160 nurses; cancer patients; hospital  clinics | Quantitative | Survey | n/a | 53% of patients experiencing significant fatigue at least daily, and 80% at least monthly on both sides of the Atlantic. In other respects, such as duration of bouts of fatigue, how much and with whom patients discuss their fatigue symptoms, and economic impacts of fatigue, there are significant differences. |
| **COMMUNICATION & EDUCATION APPROACHES** | | | | | | |
| **Dowling, S.**  **et al. (2005)** | To ascertain what further training Irish GPs want in this discipline, and how such training might be delivered. | GPs (n=32) | Mixed methods (Delphi technique) | Survey (incl. open-ended items) | n/a | GPs expressed the need for more training in the use of the syringe driver, newer treatments for pain and other symptoms, issues relating to polypharmacy, non-drug therapies, counselling and communication. Factors preventing GPs from availing themselves of further training include practice workload, difficulties in obtaining locum cover, distance and cost of courses, loss of personal time, and interference with family life. GPs would like further training to be multidisciplinary and practically orientated, and to include case discussions and workshops. |
| **Dowling, S.**  **et al. (2005)** | To evaluate the training in palliative care received by GPs completing vocational training in Ireland. | Final-year GP trainees (n=51) in Irish GP training schemes (n=10). | Quantitative | Survey | n/a | Teaching in the GP training schemes was formal and comprehensive. Over 40% admitted having no confidence in use of the syringe driver, management of stoma problems, bereavement in children, and euthanasia; 57% indicated that further practical training in palliative care was of high priority for them. Trainees indicated that dealing with their own emotions was a significant source of stress. |
| **Davidson, R. et al. (2005)** | To identify patient satisfaction with regard to various aspects of communication and perceived quality of care. | Cancer patients (n=339) | Quantitative | Survey | Patients’ views of cancer services (NHS-developed) | Key findings are that there are a number of issues with regard to information and communication which can be clearly improved within Northern Ireland cancer services. The paper concludes that patient-professional communication should be tailored to meet individual need. |
| **Dowling, S.et al. (2003)** | To establish the current status of undergraduate teaching in palliative care in this important discipline in Irish medical schools | Medical schools (n=5) | Quantitative | Survey | n/a | Identification of the relevant teachers proved to be difficult. In no school is the teaching centrally co-ordinated. All five medical schools have specific time dedicated to the teaching of palliative care; in two cases this is one day or less. All schools covered most of the topics outlined in the curriculum. The majority of the teaching is by didactic lecture; there is some use of group discussion and case studies. |
| **McIlfatrick, S.J. et al. (2003)** | To facilitate a strategic approach to cancer nursing research by identifying the research priorities of cancer nurses. | Nurses (n=60) | Mixed methods (Delphi technique) | Survey (incl. open-ended items) | n/a | The top priority areas identified were psychosocial issues, for example communication and information needs; professional issues relating to nurse burnout, stress and nurse-led care; and context of care issues including continuity of care. |
| **Donaghy, K. et al. (2002)** | To collect, analyse and summarize information on how team members perceive teamworking; compare team members' perceptions after a teambuilding workshop; and to evaluate the longer term effect of this training on the team. | Practitioners (n=67) | Mixed methods | Survey (incl. open-ended items) | n/a | A perception of understaffing was noted as being one of the largest negative influences on teamwork whereas the setting and maintaining of agreed team objectives and having sufficient education opportunity were positive influences. Although teambuilding sessions appear to have the potential to produce the desired benefits, they should not be initiated at a time when staff already feel anxiety over their workload. |
| **Timmins, F. et al. (2002)** | To examine reported stress in 12 areas commonly reported to cause stress to nursing students. | Nursing students (n=110) | Quantitative | Questionnaire | n/a | Stress exists for students in both the clinical and academic aspects of the programme. Financial constraints and academic-related concerns emerged as the most stressful areas for the students. A third of the students reported that relationships with teachers and staff on the ward cause some degree of stress. Five factors emerged as sources of stress. Firstly, 'academic' stress factors. The second and third components concern relationships, the former involving teaching-related staff, and the latter involving the clinical experience. |
| **SPECIFIC GROUPS (ID)** | | | | | | |
| **Dodd, P., et al. (2005)** | (a)To examine the type of experience  people with intellectual disabilities have following  bereavement, & (b) to assess levels of staff training and support as perceived by senior managers. | Service  agencies; care staff (n=60) | Mixed methods | Survey (incl. open-ended items) | n/a | There appears to be cultural differences in the experience of bereavement for people with intellectual disabilities. This is important in the context of staff training and policy development in grief and bereavement. |
| **McEvoy, J.**  **et al. (2005)** | To explore relatives’ perceptions of the grieving-process, concept of death and reactions to bereavement for their intellectually disabled family member. | Relatives (n=38) | Quantitative | Questionnaire | n/a | Many respondents reported post-bereavement reactions of adults with intellectual disabilities typical of the general population. Although the majority of respondents perceived their relative as not understanding the concept of death they were supportive of death education, did not view lack of knowledge as a barrier to grieving and were optimistic about supporting their relative following bereavement. Respondents with behaviour-ally challenging family members were less agreeable to death education and less optimistic about their ability to support. |
| **MacHale, R.**  **et al. (2002)** | To investigate the impact of bereavement on people with learning disability. | Adults (n=20) with learning disabilities & control gp. | Quantitative | Survey | Psychiatric Assessment Schedule for Adults with a Developmental Disability (PAS-ADD); Aberrant Behavior Checklist (ABC) | With regard to psychiatric disturbance, significant differences were found for 'neurotic disorder' and for 'organic condition' among the bereaved group on the Psychiatric Assessment Schedule for Adults with a Developmental Disability (PAS-ADD). No difference was found for 'psychotic disorder' on the PAS-ADD. As regards challenging behaviour, significant differences were found for 'irritability', 'lethargy' and 'hyperactivity' among the bereaved group on the Aberrant Behavior Checklist (ABC). No differences were found for 'stereotypy' or 'inappropriate speech' on the ABC. |
| **COMPLEMENTARY & ALTERNATIVE MEDICINE/INTERVENTION** | | | | | | |
| **Dunwoody, L. et al. (2002)** | To explore patients' experiences of aromatherapy using of a focus group interview | Cancer patients | Qualitative | Focus group interviews | n/a | Eight themes emerged from the analysis, six of which have been acknowledged to some extent by previous authors: de-stressing effects of aromatherapy, the counselling role of the aromatherapist, aromatherapy as a reward, patient empowerment, communication through touch, and negative aspects of the service. Two apparently new themes emerged concerned with security of context (where the aromatherapy took place) and preconceived perceptions of the value of aromatherapy as a treatment of cancer patients. |
| **Wright, S.**  **et al.**  **(2002)** | To explore clients’ perceptions of the benefits of reflexology interventions on their QoL. | Clients who received intervention (n=47) | Qualitative | Retrospective clinical case note review | n/a | Reflexology interventions were perceived to impact positively upon clients' levels of impairment and functional status, including physical and psychological function, with implications for general health perceptions. |
| **BEREAVEMENT** | | | | | | |
| **Gallagher, M.**  **et al. (2005)** | To evaluate bereavement counselling provided by a voluntary charity from the perspective of clients. | Counsellors (n= 89) | Quantitative | Survey | n/a | Paraprofessional voluntary counsellors can provide a counselling service to bereaved people that satisfies the majority of clients who report positive experiences in counselling and post-counselling outcomes, and generally rate their counselling as helpful. |
| **SERVICE DELIVERY** | | | | | | |
| **Ling, J. (2005)** | To assess the current level of input from community-based clinical nurse specialists in palliative care into nursing homes in the Republic of Ireland. | community-based clinical nurse specialists in palliative care (n=63) | Quantitative | Survey | n/a | All respondents had undertaken work with nursing homes. The main focus of interactions with nursing homes was on pain and symptom management and this was often provided by telephone. The majority of nurses were involved exclusively in care of patients with cancer, although 40% of respondents cared for patients with non-malignant diseases. |
| **McDonnell, M.**  **et al. (2002)** | To investigate registered nurses' perceptions of palliative care in district general hospitals. | Nurses (n=263) | Quantitative | Questionnaire | n/a | 70% of general ward nurses believe that managing care of the dying is an integral part of hospital care. However, their dissatisfaction with the care they give is reflected in the mere 8% who consider hospital an ideal setting for patients who are dying. A minority feel confident in discussing death and dying with the patient. Barriers to optical palliative care on general wards were perceived as lack of appropriate education and training, work pressures, and lack of support from medical colleagues and managers. |
| **Sullivan, K.A.**  **et al. (2005)** | To ascertain nurses’ experience of working alongside an HAH (Hospice At Home) service. | District nurses (n=162) | Quantitative | Questionnaire | n/a | The findings support the idea that an HAH service can enable patients with advanced progressive disease to be cared for at home. While the findings of this study cannot be generalized, they add to the growing body of research about HAH services in palliative care. |
| **DEATH & DYING** | | | | | | |
| **Tiernan, E.**  **et al. (2002)** | To examine the relations between depressive symptoms and desire for early death (natural or by euthanasia or physician-assisted suicide) in terminally ill patients with cancer being cared for by a specialist palliative care team. | 142 terminally ill patients with cancer | Quantitative | Questionnaire | HADS | Only 2 patients expressed a strong wish for death by some form of suicide or euthanasia. 120 denied that they ever wished for early release. The desire for early death correlated with depression scores. Depressive symptoms were common in the whole group but few were on antidepressant therapy. |
| **Tiernan, E. et al. (2002)** | To explore patients’ preferences for place of death. | Patients (n=191) referred to a Hospice Home Care Team | Mixed methods | Survey | n/a | Over 80% expressed a preference to die at home. Almost 50% of those who expressed a preference for a home death actually died there. Of those admitted to hospital or hospice to die, uncontrolled symptoms and inability of family to cope were the main reasons precipitating admission. On closer assessment, it was felt that a significant minority of those admitted could have been cared for at home. |
