# Supplementary material for: Palliative care research on the island of Ireland over the last decade: a systematic review and thematic analysis of peer reviewed publications
Source: BMC Palliat Care. 2013 Sep 4;12:33. doi: 10.1186/1472-684X-12-33 (PMC3848123; doi:10.1186/1472-684X-12-33)
Supplement: Additional file 2: Table S2 — Published palliative care research on the island of Ireland (2006–2012). [file 1472-684X-12-33-S2.doc]

**Table 2. Published palliative care research on the island of Ireland (2006-2012)**

| **Author, Year** | **Aim** | **Sample/ Setting** | **Research Design** | **Method** | **Outcome measures** | **Key Findings of the Study** |
| --- | --- | --- | --- | --- | --- | --- |
| **THEME: BEREAVEMENT** | | | | | | |
| **McGuinness, B. et al. (2011)** | To evaluate an innovative bereavement support group established in a hospice setting | Participants (n=5) | Quantitative | Questionnaire | Texas Revised Inventory of Grief (TRIG); Adult Attitude to Grief (AAG) scale | This article explores some of the issues and dilemmas raised by evaluation in this context and discusses the importance of evaluation to developing a sound evidence base for innovative bereavement support work. |
| Tracey, A. (2011) | To explore the experiences of women in Ireland who were bereaved of their mother in early life, and the lifelong impact of this loss. | Participants (n=26) | Qualitative | Interviews | n/a | The death of participants’ mothers had a profound impact throughout these women’s childhoods and adult lives. They described the silence surrounding the death, their hunger for information about their mother, their coping strategies, their yearning for a mother figure, particularly in adolescence and when they married and gave birth themselves, and the effects on their own parenting capacity. A strong theme was their wish to speak publicly about their experiences. |
| **Roberts, A. et al. (2010)** | To evaluate a bereavement information evening (BIE) as part of an adult bereavement support service | Hospice (n=78 service users) | Quantitative | Questionnaire | Texas Revised Inventory of Grief (TRIG) | BIE may provide mid-level support for people who need more than an information leaflet but not require more intensive one-to-one support. BIE may also provide access to more intensive bereavement services if necessary. |
| **Agnew, A. et al. (2010)** | To identify and review bereavement measurement tools to determine their suitability for use within bereavement services and hospice settings. |  | Systematic Review | n/a | n/a | From 59 full-text papers appraised, 10 measurement tools were analysed in detail. Some tools had been tested on specific populations which limited transferability to specialist palliative care settings; some lacked adequate theoretical links and were not effective in discriminating between normal and complicated grief reactions; and some lacked clear evidence of validity or reliability. |
| **Mc Guinness, B. (2009)** | To explore Irish organisations’ policies and procedures for supporting bereaved employees. | Private, public & local government organisations (n=34) | Quantitative | Questionnaire | n/a | While all the organisations had experienced employee-related bereavement within the previous 12 months, only four organisations had any written policy. The provision of compassionate leave varied widely and was often left to the discretion of line managers. |
| **Agnew, A. (2009)** | To gain a deeper understanding of service user experiences of an adult bereavement group in a hospice setting in Northern Ireland | Service users (n=7) | Qualitative | Individual interviews | n/a | Two main themes emerged from the data: (1) how service users felt through their experience of attending the bereavement group meetings; and (2) the language and presentation required for an appropriate bereavement group information leaflet. |
| **Agnew, A. et al. (2008)** | To explore the experiences of partners bereaved through cancer. | Bereaved partners (n=10) | Qualitative | Individual interviews | n/a | Four themes emerged: cancer journey; impact of bereavement; process of adjustment and change; and experience of support services. Findings highlight key skills, knowledge and values that should be adopted by healthcare professionals involved in the provision of care to patients and families. Informal support networks were found to be the most valued source of help. |
| **Roberts, A. et al. (2008)** | To evaluate a hospice-based bereavement support service. | Hospice (n=243 service users) | Quantitative | Questionnaire | Texas Revised Inventory of Grief; Santa Clara Strength of Religious Faith | Majority of service users were satisfied with the bereavement support service offered by the hospice. Some barriers to service delivery were also identified. |
| **Walsh, T. et al. (2008)** | To examine a hospital-based bereavement | Acute hospital (n=339 bereaved next-of-kin) | Mixed methods | Case review; questionnaire; focus group interview s | n/a | Service is being used appropriately in that the outreach contact and information provision is welcomed. High proportions of those who described the death as sudden / unexpected made contact with the service further. |
| **O'Donohoe, S. et al. (2006)** | To examine the experiences of bereaved people interacting with newspaper staff when they placed In Memoriam notices in local newspapers. | Newspaper employees (n=10) | Qualitative | Interviews | n/a | Newspaper employees engaged in philanthropic emotion management when dealing with bereaved customers. |
| **THEME: COMMUNICATION APPROACHES & EDUCATION** | | | | | | |
| **Lowney, A.C. et al. (2011)** | To examine the use of blogging by a patient receiving palliative care as a form of communication. | Specialist palliative care services; patient with pontine glioblastoma multiforme | Qualitative | Case study | n/a | This case is noteworthy for a variety of reasons. It brought hospice staff into unfamiliar territory. It challenged them to see social media as a method of communication in life-limiting illness. It provided a rare insight into the patient’s perception of hospice care. it allowed relative anonymity. It provided a fascinating account of each stage of illness. It offered a lasting legacy for the patient’s family & friends. The blog also received media attention. |
| **Callinan, J. et al. (2010)** | To understand the information needs of staff with regard to a new library and information service. To identify current access to and levels of skills in information literacy and ICT. To ascertain the need for training in those skills. | Hospice (n=48 staff including nurses, doctors/ consultants, HR, volunteers, other health care professionals) | Quantitative | Questionnaire | n/a | Assistance with obtaining journal articles was rated most highly by respondents as being an important service. 83% indicated that they did not have access to online heath databases. Small group classes were considered the preferred method of providing training. Afternoons were also considered more convenient for visiting the library. |
| **McIlfatrick, S. et al. (2010)** | To explore the educational needs of palliative link nurses. | N=80 | Quantitative | Questionnaire | n/a | Education for palliative care link nurses needs to incorporate strategies to develop competence and confidence in practice (knowing how) alongside skill-based learning (knowing that). |
| **Johnston, G. (2007)** | To evaluate a one year training scheme for Macmillan Nurses. | Trainees (n=8), Practice-Based Facilitator (n= 7), other staff (n=6) | Qualitative | Interviews | n/a | Mainly positive. Trainees were perceived to be adequately prepared to adopt the role of a CNS on completion of the scheme. Some participants thought that more theory on symptom control and communication skills and a placement in a specialist palliative care centre should also be included. |
| **THEME: COMPLEMENTARY & ALTERNATIVE MEDICINE/INTERVENTION** | | | | | | |
| **Chang, K.H. et al. (2011)** | To investigate the prevalence and predictors of Complementary and Alternative Medicine (CAM) use among cancer patients and non-cancer volunteers, and to assess the knowledge of and attitudes toward CAM use in oncology among health care professionals. | Outpatient and inpatient settings; cancer patients (n=219) and non-cancer volunteers (n=301); health care professionals (n=156) | Quantitative | Survey | n/a | The overall prevalence of CAM use was 32.5%. Female gender, younger age, higher educational background, higher annual household income, private health insurance and non-Christian were factors associated with more likely CAM use. Most health care professionals thought they did not have adequate knowledge nor were up to date with the best evidence on CAM use in oncology. Health care professionals who used CAM were more likely to recommend it to patients. |
| **THEME: DEATH & DYING** | | | | | | |
| **Ryan, K. et al. (2011)** | To explore how staff managed communication about death and dying with people with ID. | Community group homes, psychiatric hospitals, residential/ activity/sheltered work centres, general hospital & hospices; health care professionals, management & household staff (n=91) | Qualitative | Focus group interviews | n/a | Despite the general societal move toward conditional open awareness, participants in this study rarely discussed death and dying with people with ID who had life-limiting illnesses. Participants were strongly motivated to provide quality care and were willing to consider alternative approaches to communication if this would benefit people with ID. |
| **McCarthy, J. et al. (2010)** | To determine the public's understanding of and views about a range of ethical issues in relation to death and dying | 667 adult individuals | Quantitative | Phone survey | n/a | The general public are unfamiliar with terms associated with end-of-life care. Although most want to be informed if they have a terminal illness, they also value family support in this regard. Most of the respondents believe that competent patients have the right to refuse life-saving treatment. Most Irish people are more concerned about the quality of their dying than death itself. Religious commitment is important to most Irish people. |
| **Donnelly , S.M. et al. (2010)** | To explore relatives’ experience of the moment of death in a tertiary referral hospital. | Relatives (n=24) | Qualitative | Individual interviews | n/a | Four themes emerged: hospital as a place to die; quality of communication; location within hospital; and attributes of staff. |
| **Donnelly, S.M. et al. (2009)** | To examine the moment of death in a SPCU and in maximizing recall by interviewing relatives early in their bereavement. | Specialist palliative care unit; relatives (n=29) 20 families | Qualitative | Individual interviews | n/a | Participants provided a rich and detailed description of the moment of death often with humour. Additional themes were the importance of vigil; qualities of the staff; value of ritual and prayer and the environment of the SPCU. We cannot hear the stories of the dead. We can try to hear the stories of those who have witnessed dying. |
| **Donnelly, S.M. et al. (2006)** | To enquire into the phenomenon of the moment of death at home as experienced by the lay carer. | Primary carers (n=10) | Qualitative | Individual interviews | n/a | The first of three major themes describes the experience of the moment of death with particular reference to breathing and mystery. The second theme of relationship encompasses the characteristics of the carer, gender, humour, and the patient's personality. Finally, the role of the professional as guide is explored. |
| **THEME: SERVICES & SETTINGS** | |  |  |  |  |  |
| **Darker, C. et al. (2012)** | To provide baseline data on chronic disease management (CDM) provision in Irish general practice (GP). | GPs (n=360) | Quantitative | Survey | Use of Chronic Care Model Elements Survey; A Survey of Primary Care Physicians | The majority of GPs reported significant changes are needed in the Irish health care system to make CDM work better. Small numbers of routine clinical audits are being performed. Irish GPs use evidence based guidelines for treatment of diabetes, asthma / COPD and hypertension, to the same extent as international counterparts. Barriers to delivering chronic care include increased workload, lack of appropriate funding, with GPs interested in targeted payments. |
| **Afzal, N. et al. (2011)** | To examine the quality of end-of-life care received by patients with & without dementia on acute medical wards. | Acute medical wards; patient notes (n=75) | Qualitative | Retrospective clinical case note review | n/a | Dementia patients were significantly less likely to be referred to palliative care interventions, to be prescribed palliative drugs & to have carers involved in decision making. |
| **Agnew, A. et al. (2011)** | To measure the satisfaction of District Nurses (DN) & service users with a multi-visit (MV) service. | District Nurses (n=21), service users (n=22) | Quantitative | Phone survey | n/a | All the DN and service users reported that the MV service enabled patients to continue to be cared for in their own homes. |
| **Blaney, J.M. et al. (2011)** | To establish factors that influence and contribute to the death of patients with cancer in acute hospitals in Northern Ireland | 16 acute hospitals; 695 adults patients with cancer | Qualitative | Retrospective clinical case note review | n/a | 3 main reasons for acute hospital deaths were uncovered. (1) 26% of patients were diagnosed with cancer during their last hospital admission. (2) Patients were very ill with nearly 79% admitted as an emergency, requiring medical attention as a result of cancer-related and urgent physical symptoms. (3) Despite 38% of patients specifically requesting discharge to their usual residence, hospice or other hospital, this was not achieved for various reasons. |
| **Bracken, M. et al. (2011)** | To assess the utility of three needs assessment/ dependency tools for use with patients in community-based palliative care services. | CNSs (n=22) | Mixed methods | Assessments; individual and focus group interviews (n=2) | Vale prioritization tool; Graves & Payne (2007) and Birch et al. (1997) dependency tools | The Vale prioritization tool appeared to be the most useful for prioritizing patient need and managing workload. Three themes emerged from the interviews: difficulties with routine administration, points of divergence between the two dependency tools, and workload concerns. Findings raise questions about the overall utility and practical application of these kinds of tools with community-based palliative care patients. |
| **Casey et al. (2011)** | To explore the factors that influence the provision of good end-of-life care for older people in acute and long-stay care settings in Ireland | Acute and long-stay care settings; staff (n=33) | Qualitative | Interviews | n/a | Factors that influence provision of end-of-life care in Ireland were identified. The core category was 'dying well'. The potential to 'die well' was influenced by three factors, namely philosophy, culture and organisation of care, knowing the person and physical environment and resources. This study identified the factors that influence the provision of good end-of-life care for older people. |
| **McCormack, R. et al. (2011)** | To establish the reasons for phlebotomy and evaluate the usefulness of blood testing in the palliative setting. | Inpatients (n=65) with malignant & non-malignant diagnosis | Quantitative | Questionnaire | n/a | The top three diagnosis were malignancy of bowel, ovary, and prostate respectively. The top three reasons for venepuncture were to manage medications, establish the need for blood transfusion, and guide management of sepsis. 30% of phlebotomy sessions changed management, 40.7% ruled in an important diagnosis, and 86% ruled out an important diagnosis. 48% of phlebotomy sessions had at least one type of tests “added on” that in hindsight was unnecessary. |
| **Ó Céilleachair, A. (2011)** | To investigate time trends in place of death for colorectal cancer. | 10,175 colorectal cancer deaths | Quantitative | Database | n/a | Nearly half (49%) of deaths occurred in acute hospitals, 29% at home, 13% in hospices and 7% in nursing homes. Hospital deaths were unchanged over time. Hospice deaths rose from 6% in 1994 to 17% in 2003. Home deaths decreased significantly, but only in health boards with hospices. Nursing home deaths rose significantly in areas without hospices. |
| **Devlin, M. et al. (2010)** | To explore the role of home-care workers in palliative and end of life care in the community. | Community nurses (questionnaire, n=69; focus group, n=6) | Mixed methods | Questionnaire; focus group | n/a | Participants identified the need for additional support in the provision of end-of-life care, specifically during out-of-hours. Various restricting factors such as training deficits, need for support and supervision and communication issues were also identified. |
| **McLean, S. et al. (2010)** | To report findings of a systematic review of current practice and attitudes towards the use of primary thromboprophylaxis for patients with advanced cancer in the palliative care setting. | Acute, outpatient and inpatient SPC settings; patients (n=198) | Systematic review | Studies examined (n=8) | n/a | Patients find LMWH (low-molecular-weight-heparin) acceptable, particularly patients who experienced a sudden decline in performance status. Reluctance to prescribe LMWH is based on physicians’ concerns regarding negative impact on quality of life, and lack of evidence specific to the palliative care setting. LMWH prophylaxis should be implemented in patients with a previously good performance status who have a transiently increased of VTE and no contraindications. |
| **Sharp, L. et al. (2010)** | To investigate (time) trends in where cancer patients die to inform decisions about how healthcare should be organised to support those in need of end-of-life care. | 18,078 death certificates | Quantitative | Database | n/a | 53% of deaths occurred in an acute hospital, 29% at home, 12% in hospices and 4% in nursing homes. Hospice deaths rose from 7% in 1994 to 15% in 2003, falling slightly in 2004-05. Hospital deaths were unchanged over time, but were more common in areas without hospices. Home deaths decreased significantly Nursing home deaths rose significantly. These trends were not explained by temporal changes in the age-sex distribution of deaths. |
| **Curry, C. et al. (2009)** | To explore and address the palliative care education needs of staff working in two nursing homes. | Registered nurses (n=4) and care assistants (n=8) in two nursing homes | Mixed methods | Questionnaire; focus group interviews | n/a | Providing palliative care education using a practice development framework enables and empowers staff to provide greatly improved end of life care to residents. ‘Knowing the person’ is an important aspect of caring for residents in nursing homes and life story work can be used to enhance their care. |
| **Roche-Fahy, V. et al. (2009)** | To explore the lived experience of nurses who provide comfort to palliative care patients in an acute setting in a small urban hospital. | Acute hospital; general non-specialised nurses (n=12) | Qualitative (Gadamerian hermeneutic phenomenology) | Individual interviews | n/a | Four main themes emerged as central to nurses’ experiences: time needed to provide comfort; emotional labour of providing comfort; holistic approach involved in providing comfort; and education and expertise and their role in providing comfort. |
| **Devlin, B. et al. (2008)** | To evaluate a domiciliary blood transfusion home service for palliative care patients. | Patients (n=11) who had received a domiciliary blood transfusion and who were in the palliative stage of their illness. | Quantitative | Phone survey | n/a | Domiciliary blood transfusions delivered in the home helped to avoid unnecessary hospital admissions and improved QoL of patients & carers. |
| **Hasson et al. (2008)** | To explore link nurses’ views and experiences regarding development, barriers and facilitators to the implementation of the role in palliative care in the nursing home. | Nursing homes (n=10); link nurses (n=14) | Qualitative | Focus group interviews | n/a | The link nurses system shows potential to enhance palliative care within nursing homes. Facilitators to implementing the role included external support, monthly meetings, access to a resource file and peer support among link nurses themselves. Barriers included lack of management support, a transient workforce and the lack of adequate preparations for link nurses. |
| **Lucey, M. et al. (2008)** | To (a) perform a systems analysis of the process by which patients under the care of a specialist palliative home care obtained medications and factors that delay this process & (b) document the prevalence of each factor in the system causing delay. | Specialist palliative home care service; GPs (n=111); pharmacists (n=57); patients (n=22) | Mixed methods | Questionnaire; prospective observational study | n/a | Main factors causing delay were: medications not being in stock in pharmacies, medications not being available on state reimbursed schemes and inability of patients and carers to courier medications. |
| **Twomey, F. et al. (2008)** | To compare the ability of 5 professional groups to estimate the survival of patients admitted to a SPC unit. | Clinicians (n=40) | Quantitative | Survey | n/a | No group accurately predicted the length of patient survival more than 50% of the time. Nursing and junior medical staff were most accurate while care assistants were least accurate. When in error, senior clinical staff tended to under-estimate survival. |
| **Waldron, M. et al. (2008)** | To assess the palliative care education received and consequently cascaded by designated nursing home staff. | Private nursing home; nursing staff (n=30) | Quantitative | Survey | n/a | There was a high satisfaction with course content, facilitation and benefits accrued from participation. Many respondents had not commenced cascading training within their nursing homes to lack of time and competing mandatory demands. |
| **Bailey, M.E. et al. (2007)** | To develop and evaluate guided group reflective practice in | Hospice; palliative care nurses (n=8) | Qualitative | Interviews | n/a | Group evaluation of the project is discussed under the following themes: understanding the process of reflective practice; the value of keeping a reflective diary; guided group reflection and moving forward. The introduction of guided reflection for palliative care nurses has afforded both the facilitators and the participants an opportunity to meet away from the clinical environment, and to work together, finding fresh insights to inform practice. |
| **McIlfatrick, S. (2007)** | To assess the palliative care needs of patients receiving palliative care from the perspective of patients, informal carers and healthcare professionals. | Patients & lay carers (n=24); professional palliative care providers (n=52) | Qualitative | Individual & focus group interviews | n/a | Professional providers experienced difficulty in defining the term palliative care. Difficulties in communication and information exchange, and fragmented co-ordination between services were identified. The main areas of needs identified by all participants were social and psychological support; financial concerns; and the need for choice & info. All participants considered that there was inequity between palliative care service provision for patients with (non) cancer diseases. |
| **McLaughlin, D. et al. (2007)** | To explore the bereaved caregivers’ experience of the Hospice at Home service delivered in one region of the UK. | Caregivers (n=128) | Quantitative | Questionnaire | n/a | Generally positive experiences of the Hospice at Home service. A number of suggestions were made relating to increased awareness of the service, training for staff, coordination of service delivery and bereavement support. |
| **Robinson, F. et al. (2007)** | To explore attitudes and experiences of doctors and nurses regarding cardiopulmonary resuscitation for patients with end stage illness in an acute hospital. | Acute hospital (n=16 clinicians) | Qualitative | Focus group interviews | n/a | Variation among participants in terms of how resuscitation policy and DNAR are both interpreted. Interprofessional conflict reported due to different approaches to patient care as well as some cultural differences. Ongoing staff training and education required. |
| **Kernohan, G. et al. (2006)** | To explore patient satisfaction on medical, social and therapeutic out-patient palliative care services in a hospice setting. | Hospice day care centre (n=1);  Patients (n=26) | Mixed methods | Chart review; Questionnaire | n/a | Generally positive findings. A number of suggestions for new service activities were made, and the need for education of multi-professional team members was recognised. Recognised that day hospice allowed respite care for the family. |
| **McIlfatrick, S. et al. (2006)** | To explore the nurses’ experience of a day hospital chemotherapy service in an acute general hospital & how this compared with their experience of working in an inpatient setting. | Nurses (n=10) | Qualitative | Individual interviews | n/a | Nurses viewed their experience of a chemotherapy day hospital as having both positive and negative dimensions. The positive dimensions included an increased sense of autonomy and the challenge of developing new skills, while the negative dimension included a perceived decrease in their caring role. There is a need to achieve a balance between delivering a clinical role while maintaining the centrality of the nurse-patient relationship. |
| **Mc Nicholl, M.P. et al. (2006)** | To describe the experience of staff in one acute hospital in Northern Ireland who adapted the Liverpool Care Pathway for the dying patient. | Acute hospital | Qualitative / audit | Observational case study | n/a | Key finding is that as long as patients continue to meet the criteria for LCP, some may be on the care pathway for more than the recognised average of Two days. |
| **Whittaker, E. (2006)** | To explore the level of palliative care knowledge among qualified staff delivering end-of-life care in nursing home settings, to inform the development of an appropriate education and training programme. | Nursing homes (n=48); 227 nursing staff | Quantitative | Questionnaire | n/a | Results indicated that less than half the sample had obtained formal training in the area of pain assessment and management and less than a quarter had obtained training in non-malignant conditions. Registered nurses in this study reported a lack of awareness of palliative care principles or national guidelines. Qualified nursing home staff agreed that palliative care is a valuable model for care in their setting. |
| **THEME: SPECIFIC GROUPS (CHILDREN)** | | | | | | |
| **Nicholl, H. et al. (2012)** | To explore mothers' experiences of caring for a child with complex needs | Mothers (n=17) | Qualitative | Individual interviews; documentary analysis | n/a | Caring for a child with complex needs involves the delivery of care in an inside world of the home, the world outside the home, and a “going-between” world. Caregiving were found to be 1 of 8 closely linked dimensions and included the following 4 categories: normal mothering, technical caregiving, pre-emptive caregiving, and individualised caregiving. |
| **Price et al. (2012)** | To examine the experiences of bereaved parents concerning the care provided to children who died from cancer compared to those who died from a non-malignant condition. | Bereaved parents of children who died, recruited through two regional centres | Qualitative | Individual interviews | n/a | Typically, parents of children with cancer considered care at the end of life as well resourced and responsive to their and their child's needs. In contrast, parents of children with non-malignant conditions reported under-resourced and inadequately responsive services. Although both groups of parents called extensively on military metaphors such as ‘battle’, ‘fight’ and ‘struggle’, the focus of their respective energies was different. In the one case the adversary was disease and illness; in the other it was service providers and service provision. |
| **Gilrane-McGarry, U. et al. (2011)** | To identify and describe the bereavement experiences of grandparents following the death of a grandchild and to explore their needs and supports throughout this experience. | Grandparents (n=17) | Qualitative | Individual interviews | n/a | Grandparents experience double and ‘cumulative pain’. There is a need for the complexity and intensity of the grief felt by bereaved grandparents to be recognised, acknowledged, and supported by health professionals and society. |
| **Price, J. et al. (2011)** | To explore the experiences of recently bereaved parents. | Parents (n=25) | Qualitative | Interviews | n/a | Four analytically distinct processes were identified in the responses of parents to the death of a child. These are referred to as ‘piloting’, ‘providing’, ‘protecting’ and ‘preserving’. Regardless of individual circumstances, these processes were integral to all parents’ coping, enabling an active ‘doing’ for their child and family throughout the trajectory of their child's illness and into bereavement. |
| **Quinn, C. et al. (2011)** | To elicit the views and experiences of palliative care CNSs working in the community with children and families requiring palliative care from a service primarily planned for adults. | Community palliative care clinical nurse specialists (CNSs) | Qualitative | Focus group interviews | n/a | Four key themes emerged: gaining access to the child and family; role complexities; pressures of caring; and support strategies. Provision of community children’s palliative care by CNS is complex. |
| **Kiernan, G. et al. (2010)** | To investigate physicians’ and nurses’ perceptions of psychosocial issues in pediatric oncology including their awareness of the psychosocial impact of childhood cancer on families and their knowledge and views of psychosocial interventions. | Physicians (n=6) and nurses (n=4) | Qualitative (phenomenological approach) | Individual interviews | n/a | Despite a lack of formal training in psychosocial issues, professionals identified a number of psychosocial issues associated with childhood cancer including effects for family members. They recognised the value of formal intervention for children, families and themselves. Findings suggest the need for more formal training on psychosocial issues for medical and nursing staff. |
| **McCloskey, S. et al. (2010)** | To explore the experiences of stress in nurses providing children’s palliative care. | Children’s hospice nurses (n=9), community children’s nurses (n=7), children’s nurse specialists (n=2) | Qualitative | Focus group interviews | n/a | Four core themes emerged: work demands (emotional load, ethical conflicts, constraints to the delivery of good care, limited resources, administration & living and working in the same community); relationships; maintaining control; and support and roles (managerial support and role ambiguity). Implications for how individuals and organisations may reduce impact for nurses in various settings are considered. |
| **Meyler, E. et al. (2010)** | A systematic review of family-based psychosocial interventions with pediatric oncology populations. | Studies (n=21) meeting inclusion criteria | Systematic review | n/a | n/a | Findings illustrate that multiple family members are integrated into interventions for childhood cancer in several ways. Considerable variations were observed regarding the family member combinations targeted and the intervention modalities employed. A minority of interventions met the criteria for empirically validated treatments; however; evidence of beneficial outcomes was observed across the majority of interventions reviewed. |
| **Clarke, J. et al. (2007)** | To explore the experiences of professional carers’ in providing pediatric palliative care to children with life-limiting conditions. | Professional groups providing hospital and/or community-based care in the voluntary and statutory areas. | Mixed methods | Questionnaire; focus group interviews (n=15) | n/a | The humanity of professional carers’ caring reality may be articulated through three themes: clarity of definition and complexity of engagement; seeking to deliver a palliative care service; and the emotional cost of providing palliative care. Further analysis highlights a work-life experience of skilled and emotional engagement with children, and their parents, in complex processes of caregiving and decision-making. |
| **McCluggage, H.L. et al. (2006)** | To identify the symptoms experienced by life-limited children which cause anxiety to staff working in children’s hospices. | Hospice administrators (n=10); nurses (n=18); doctors (n=10) | Quantitative | Questionnaire | n/a | More than 70% of all staff groups felt that identifying the symptom correctly caused more anxiety than treating identified symptoms. Doctors & nurses perceive seizures, pain management & vomiting as the most troublesome symptoms for children with life-limiting conditions. |
| **O’Leary, N. et al. (2006)** | To examine the experience of an adult palliative care service providing palliative care to children. | Case review of children (n=20); questionnaires (11 nurses; 3 medical staff); focus group (n=8 nursing/ medical staff) | Mixed methods | Case review; Questionnaire; Focus group interview | n/a | Main themes highlighted were staff competence, staff stress, uncertainly of prognosis, resource implications and co-operation with other teams. Some key challenges highlighted for an adult palliative care team providing paediatric palliative care. |
| **THEME: SPECIFIC GROUPS (COPD)** | | | | | | |
| **Hynes, G. (2012)** | To explore the experiences of informal caregivers providing care in the home to a family member with COPD. | Family caregivers (n=11) of individuals with COPD | Qualitative | Interviews | n/a | Six core themes emerged including 'then and now' reflecting caregivers' sense of loss and enmeshment with the illness experience and burden. The caregivers' experience of illness burden included symptom, cultural and lifeworld meanings. Relationships between formal health care and healthcare professionals were rendered difficult by their perceived failure to look beyond acute exacerbations as discrete events rather than integral to the illness trajectory as a whole. |
| **Casey, D. et al. (2011)** | To develop a structured education programme for clients with COPD. | COPD patients (n=16); health professionals (n=25) | Qualitative | Interviews | n/a | Findings indicate that nurses working in primary care have an important role in informing the development of SEPRP (Structured Education Pulmonary Rehabilitation Programme) as well as having a key role in their delivery.  They have the potential to work in empowering ways with COPD patients and the PRINCE (Pulmonary Rehabilitation In Nurse-led Community Environments) SEPRP is just one example of how this can be put into practice. |
| **Hasson, F. et al. (2009)** | To explore the experiences of palliative care that bereaved carers had while providing care to a dying loved one with COPD. | Carers with advanced COPD (n=9) | Qualitative | Individual interviews | n/a | Three themes emerged: the impact of the caring experience, the lack of support services, and end-of-life and bereavement support. Carers experienced carer burden, lack of support services, need for palliative care, and bereavement support. |
| **Spence, A. et al. (2009)** | To explore professionals’ perceptions of palliative care and facilitators and barriers to the delivery of such care for patients with advanced COPD. | Health and social care professionals (n=23) | Qualitative | Individual and focus group interviews | n/a | Care of patients with COPD is focused upon the management of symptoms, with emphasis focused predominantly on an acute model of care. Key barriers towards the delivery of palliative care included the reluctance to negotiate end-of-life decisions and a perceived lack of understanding among patients and carers regarding the illness trajectory. Consequently the delivery of palliative care was viewed as a specialist role rather than an integral component of care. |
| **Hasson, F. et al. (2008)** | To explore the potential for palliative care among people living with advanced chronic obstructive pulmonary disease (COPD). | Patients with a diagnosis of advanced COPD (n=13) | Qualitative | Individual interviews | n/a | Participants raised concerns about the unknown trajectory of the illness and reported unmet palliative care needs with poor access to palliative care services. Research suggests that needs for palliative care be extended to all (regardless of diagnosis), with packages of care developed to target specific needs. |
| **Spence, A. et al. (2008)** | To explore the specific care needs of informal caregivers of patients with advanced COPD. | Active family caregivers (n=7) | Qualitative | Individual interviews | n/a | Family caregivers provide direct care with little support and assistance. There were knowledge deficiencies among caregivers relating to the COPD illness trajectory and little awareness of the potential of palliative care. Family caregivers need social and professional support/interventions while caring for a patient at home. |
| **THEME: SPECIFIC GROUPS (DEMENTIA)** | | | | | | |
| **Connolly, S. et al. (2012)** | To estimate the economic and social costs of dementia in Ireland in 2010 | People with dementia | Quantitative | Database analysis | n/a | The total baseline annual cost was found to be over €1.69 billion, 48% of which was attributable to the opportunity cost of informal care provided by family and friends and 43% to residential care. |
| **Gallagher, D. et al. (2011)** | To examine the relationship between self-efficacy for dementia-related tasks and symptoms of burden and depression in caregivers. | 84 patient/caregiver dyads with Alzheimer's disease | Quantitative | Assessments; questionnaires | DSM-R IV; NINCDS-ADRDA criteria; Mini Mental State Examination; Eysenck personality questionnaire; Zarit Burden Inventory; CESD-10; Lubben social network scale; Neuropsychiatric Inventory (NPI); Disability Assessment for Dementia scale (DAD); 10-item self-efficacy scale; Brief COPE | 33% of caregivers reported significant depressive symptoms. Caregiver burden was predicted by self-efficacy for symptom management, neuroticism, patient function and neuropsychiatric symptoms while caregiver depression was predicted by self-efficacy for symptom management, caregiver educational level, neuroticism, emotion-focused coping, dysfunctional coping and patient function. |
| **Pierce, M. et al. (2012)** | Planning dementia services: New estimates of current and future prevalence rates of dementia in Ireland. (paper provides new estimates of dementia prevalence at a national and local level in Ireland). | People with dementia | Quantitative | Database analysis | n/a | It is estimated that there were 41,740 people with dementia in Ireland in 2006. Estimates show that there are clear regional differences in prevalence of dementia across Ireland. |
| **THEME: SPECIFIC GROUPS (HEART FAILURE)** | | | | | | |
| **Hughes, C. et al. (2012)** | To determine the dietary adequacy of CHF patients compared with Dietary Reference Values, to compare the nutritional intake and status of CHF patients to a healthy comparison group, and finally to determine whether nutritional intake and status depended on New York Heart Association (NYHA) functional class. | Patients with chronic heart failure (n=39) & a comparison group of health participants  (n=27) | Quantitative | Assessments | n/a | Overall 73% of the CHF patients were consuming less than recommended energy intakes, and more than 50% of these patients were also consuming less than recommended vitamins and minerals. |
| **O’Leary, N. et al (2009)** | A comparative study of the palliative care needs of heart failure and cancer patients. | 50 HF patients (n=50); cancer patients (n=50) | Mixed methods | Interviews; questionnaire | Nottingham Extended Activity Daily Living Scale (NEADL); Edmonton Symptom Assessment Scale (ESAS); HADS; Short Form-36 | Both patient cohorts were statistically indistinguishable in terms of symptom burden, emotional wellbeing, and quality-of-life scores. HF patients had good access to community and social support. HF patients particularly valued the close supervision, medication monitoring, ease of access to service, telephone support, and key worker provided at the HF unit. A small subset of patients had unmet PC needs. |
| **Ryan, M. et al. (2009)** | To describe patients' experiences of living with advanced heart failure | Patients with advanced (NYHA classes III–IV) heart failure (n=?) | Qualitative | Individual interviews | n/a | Four main themes emerged: Living in the Shadow of Fear; Running on Empty; Living a Restricted life; and Battling the System. The experience of living with advanced heart failure was described as a fearful and tired sort of living characterised by escalating impotence and dependence. |
| **THEME: SPECIFIC GROUPS (ID)** | | | | | | |
| **Mc Evoy, J. et al. (2012)** | To investigate how individuals with ID understand and explain death and make sense of life without the deceased | Individuals with intellectual disabilities (n=34) | Qualitative | Interviews | n/a | Death comprehension was positively correlated with cognitive ability and adaptive functioning. While cause of death was predominantly associated with illness and old age, participants viewed death as final and understood that all living things die. The role of religious beliefs was also found to be important for many participants. The results support earlier findings that suggest people with ID have only a partial understanding of the concept of death leaving them vulnerable to factually incorrect thoughts. |
| **McCarron, M. et al. (2011)** | To better describe the role and timing of palliative care in supporting persons with intellectual disabilities and advanced dementia (AD). | Intellectual disability services (n=6); staff (n=?) | Qualitative | Focus group interviews | n/a | Specialist palliative care staff recognised that person-centred care delivered in intellectual disability services was consistent with palliative approaches, but staff in intellectual disability services did not consider advanced dementia care as 'palliative care'. Both groups were unsure about the role of palliative care at early stage of dementia but appreciated specialist palliative care contributions in addressing pain and symptom management challenges. |
| **Ryan, K. et al. (2011)** | To explore paid carers’ perspectives when caring for people with ID at the end of life. | Various settings (see Ryan, K. et al., 2011); health care professionals (n=64) | Qualitative | Focus group interviews (n=16) | n/a | Study describes issues which contribute to the development of staff stress when providing palliative care (e.g. situations when end of life care decision making was challenging, when staff felt ‘pushed out’ by relatives, and when staff did not have sufficient support or time to provide care or mourn the loss of service users. |
| **McCarron, M. et al. (2010)** | To understand staff perceptions of critical issues in caring for persons with intellectual disability (ID) and advanced dementia. | Persons with ID and advanced Dementia | Qualitative | Focus group interviews | n/a | Views of specialist palliative care staff. |
| **Ryan, K. et al. (2010)** | To describe the experience, confidence and attitudes of staff to the provision of palliative care to people with ID. | Staff from ID and palliative care services (n=261) | Mixed methods | Questionnaire & focus group interviews | n/a | Both palliative care and intellectual disability services staff lacked confidence in their ability to provide palliative care. Staff were challenged by perceived ‘differences’ and ‘difficulties’ in the provision of care. They endorsed a partnership approach to care but a shared desire to cooperate did not predict effective collaboration. |
| **Fahey-McCarthy, E. (2009)** | To understand care concerns with respect to supporting persons with ID and advanced dementia, and to develop, deliver, and evaluate an educational intervention with staff in ID settings and specialist palliative care services | Intellectual disability services (n=6); specialist palliative care provider (n=1) | Qualitative | Focus group interviews | n/a | Data analysis informed development of an educational intervention. Staff indicated that the educational intervention was highly valued and addressed key training concerns. They agreed that the training supported “aging in place,” and the preparation for a “good death” including support for staff, peers, and family in their grief and bereavement. An educational intervention in the form of a trainer manual was produced to support cross-service system in-service training on issues of addressing advanced dementia in persons with ID. |
| **Guerin, S. et al. (2009)** | To describe and gather preliminary psychometric data for a version of the Inventory of Complicated Grief for people with ID (CGQ-ID). | Patients with ID (n=76) | Quantitative | Assessments | CGQ-ID;  The Index of Social Competence | The final scale and subscales (Separation Distress and Traumatic Grief) showed very good internal and inter-rater reliability and distinguished between the two groups (i.e. half experienced a parental bereavement within the last 2 years and half who had not). Findings suggest that the CGQ-ID is suitable for identifying complicated grief-type symptoms among adults with ID, further research required. |
| **MacHale, R. et al. (2009)** | To explore staff perceptions of service users’ conceptualisations of death, reactions to bereavement, required levels of support and staff confidence in providing post-bereavement support. | Service users with ID (n=32); Care staff (n=42) | Quantitative | Survey | Index of Social Competence; Staff Attitude Questionnaire (STAQ) | Staff believed that service users had a good concept of death, though staff tended to overestimate levels of understanding and possibly underestimate the potential dysfunctional behaviour post-bereavement. Although staff expressed confidence in their ability to recognise grief symptoms, they were less confident in their ability to provide post-bereavement support. |
| **Dodd, P. et al. (2008)** | To examine the occurrence of symptoms of complicated grief, and to explore the relationships between complicated grief and bereavement experience | Individuals with an ID (n=76) who had experienced a parental bereavement within the previous 2 years | Quantitative | Questionnaire | Complicated Grief Questionnaire for People with ID; Bereavement History Questionnaire (adapted); Index of Social Competence | Study revealed that bereaved individuals with ID experience complicated grief symptoms following the death of a parent, with one-third of the bereaved group experiencing 10 or more clinically apparent symptoms. In addition, complicated grief symptoms were more likely to occur with higher rates of bereavement ritual involvement. |
| **Reynolds, S. et al. (2008)** | To examine the impact of a staff-training program on knowledge and confidence in supporting people with intellectual disabilities (ID) at the time of bereavement | Intellectual disability support service; staff (n=33) | Quantitative | Survey | n/a | Over the 4 weeks, there was a significant increase in confidence among the training group, with no comparable changes in the control group. Neither group showed significant changes in general support or job satisfaction. Findings suggest that the training significantly increased staff members’ confidence in their ability to respond to the challenges of support service users during bereavement. |
| **THEME: SPECIFIC GROUPS (Malignant)** | | | | | | |
| **Roulston, A. et al. (2012)** | A pilot study to evaluate an outpatient service for people with advanced lung cancer. | Patients (n=4) | Mixed methods | Interviews; assessments | Eastern Cooperative Oncology Group Performance Status Rating (ECOG-PSR), the Hospital Anxiety and Depression Scale (HADS), the EQ-VAS, and the EQ-5D | Many patients with lung cancer are symptomatic from diagnosis, and quality of life (QoL) may be maximised through the use of specialist palliative care in parallel with other treatments. Study found that preconceived ideas about a ‘Breathing Space’ attendance were replaced with positive impressions. Anxiety and EQ-VAS scores improved for all patients, and depression scores improved for four of the five patients, although no tests of significance were made. The qualitative data indicated that there were psychosocial benefits to attending the clinic. |
| **Donohoe, C.L. et al. (2011)** | Cancer cachexia: mechanisms and clinical implications. | n/a | Systematic review | n/a | n/a | Cachexia is compounded by anorexia and the relationship between these two entities has not been clarified fully. Inconsistencies in the definition of cachexia have limited the epidemiological characterisation of the condition and there has been slow progress in identifying therapeutic agents and trialling them in the clinical setting. |
| **Donohoe, C.L. et al. (2011)** | To examine the impact of young age on outcomes in esophageal & junctional cancer. | Patients (n=2129) diagnosed with esophageal carcinoma | Qualitative | Retrospective clinical case note review | n/a | Younger patients (aged less than 50 years) were more likely to be treated with curative rather than palliative intent. Multivariate analysis revealed independent factors related to difference in survival included sex, age, advanced T stage, and nodal metastases. |
| **Hegarty, J. et al. (2010)** | To compare the beneficial and harmful effects of radical prostatectomy (RP) versus watchful waiting (WW) for the treatment of localised prostate cancer. |  | Systematic review | n/a | n/a | Two trials met the inclusion criteria. After 12 years of follow up, the trial results were compatible with a beneficial effect of RP on the risks of overall mortality, prostate cancer mortality and distant metastases compared with WW but the precise magnitude of the effect is uncertain. Compared to WW, RP increased the absolute risks of erectile dysfunction and urinary leakage (based on self-administered questionnaires). |
| **Curran, D. et al. (2009)** | To investigate the quality of life of palliative chemotherapy naive patients with advanced adenocarcinoma of the stomach or esophagogastric junction treated with irinotecan combined with 5-fluorouracil and folinic acid: results of a randomised phase III trial. | Patients (n=333) | Quantitative (Randomised Control Trial) | Assessments | EORTC QLQ-C30 | The time-to-progression for IF and CF was 5.0 and 4.2 months respectively. The overall compliance rates for QL questionnaire completion were 60 and 56% in the IF and CF arms, respectively. Significant treatment differences were observed. There was a trend in favour of IF over CF in time-to-progression. The IF group also demonstrated a better safety profile than CF and a better QL on a number of multi-item scales, suggesting that IF offers an alternative first-line platinum-free treatment option for advanced gastric cancer. |
| **Donohoe, C.L. et al. (2009)** | Palliative endoscopic trans-anal resection (ETAR) of advanced rectosigmoid carcinoma. | Patients (n=14) | Qualitative | Retrospective clinical case note review | n/a | ETAR provides a convenient and safe method of palliation for patients with local symptoms of advanced rectosigmoid carcinoma. |
| **Mullane, M. et al. (2009)** | To validate the Demoralization Scale in a sample of 100 Irish advanced cancer patients. | In-patients with advanced cancer (n=76) | Quantitative | Assessments | Demoralization Scale; Beck Depression Inventory; Patient Health Questionnaire; Beck Hopelessness Scale; Schedule of Attitudes toward Hastened Death; McGill Quality of Life Questionnaire; Hunter’s Opinions and Personal Expectations Scale. | The findings show that in an Irish palliative care context, demoralization is not differentiated from depression. Additional factor analytic studies are needed to validate the Demoralization Scale. |
| **Timon, C. et al. (2006)** | To document the outcome of patients deemed at initial diagnosis to be appropriate for palliative care. | Patients (n=286) with head and neck mucosal squamous cell carcinoma (HNMSCC) | Qualitative | Retrospective clinical case note review & database analysis | n/a | One in five patients presenting with HNMSCC were deemed appropriate for palliative care at initial presentation and survived less than six months after diagnosis. More than one-third required surgical intervention, and 29 per cent never returned home. |
| **THEME: SPECIFIC GROUPS (Non-malignant)** | | | | | | |
| **O’Leary, N. et al. (2008)** | To describe Irish Specialist Palliative Care (SPC) services’ provision for, and attitudes to non-cancer patients. | Clinical managers (n=65) | Quantitative | Questionnaire | n/a | There is a mismatch between availability of palliative care services for non-cancer patients and uptake. Perceived barriers to service provision include unpredictable nature of non-cancer disease trajectory, issues with referral criteria and lack of non-cancer specific expertise. |
| **Fitzsimons, D. et al. (2007)** | To explore the palliative care needs of patients with a non-cancer diagnosis from the perspectives of the patient, their significant other and the clinical team responsible for their care. | Regional hospital; Patients (n=18) diagnosed with end-stage heart failure, renal failure or respiratory disease. | Mixed methods | Individual interviews (n=18 patients and separately n=17 significant others); focus group (n=18 clinical team) | Short Form 36 (SF36); Hospital and Anxiety Depression Scale(HADS) Questionnaire | Deteriorating health status led to decreased independence, social isolation and family burden. General resources and support were perceived as lacking. All participants expressed concerns regarding the patients’ future and some patients described feelings of depression or acceptance of the inevitability of death. An earlier and more effective implementation of the palliative care approach is necessary if the needs of patients in the final stages of chronic illness are to be adequately addressed. |
| **THEME: SPECIFIC GROUPS (Older people)** | | | | | | |
| **Ní Chróinín, D. et al. (2011)** | To investigate the effect of the death of older Irish persons on patients and staff in a 320-bed long-stay elderly care facility. | Patients (n=7); staff (n=3) | Qualitative | Individual interviews | n/a | Participants described feelings of loss, in adequacy, and the desirability for patients and family to have privacy during the dying phase. The researchers identified a four-stage model to describe the fellow patient grief reaction: (1) remembrance; (2) reflection; (3) religiosity; and (4) relations. Findings highlight the need for bereavement care programmes in elderly care units. |
| **Payne, S. et al. (2009)** | To examine the end-of-life care experiences of older people in acute care hospitals and long-stay institutions in Ireland. | Hospitals and long staff facilities (n=592); health care workers (n=35);  Older patients (n=30) | Mixed methods | Survey; interviews | n/a | Proposed a new model and framework to guide the delivery of palliative and end-of-life care to older people in institutions. Proposed a conceptual model for understanding older people’s experiences of transitions near the end of life. |
| **McDonnell, M. et al. (2009)** | To identify the palliative care education needs of registered general nurses (RGNs) and health-care assistants (HCAs) working in care of the older | HSE older care units; RGNs (n=205); HCAs (n=154) | Quantitative | Questionnaire | n/a | Specific education needs identified by RGNs and HCAs. Significant difference between RGNs and HCAs’ level of understanding of palliative care. Study identified the need to develop separate education programmes. |
| **THEME: SPECIFIC GROUPS (Parkinson’s)** | | | | | | |
| **McLaughlin, D. et al. (2011)** | To explore the caring experience of relatives with Parkinson's Disease. | Informal family caregivers (n=26) | Qualitative | Individual interviews | n/a | Results highlight the widespread burden of providing care on the emotional and physical health of the caregivers. The financial implications for providing care were outlined. From the point of diagnosis, carers did not feel health professionals integrated them within the caring journey. Since diagnosis, carers commented on the lack of continued and coordinated care plans for relatives, resulting in symptoms being mismanaged and care opportunities for relatives and carers missed. |
| **Waldron, M. et al. (2011)** | To examine the social worker’s role in the delivery of palliative care to clients with Parkinson’s disease (PD). | Community and hospice settings; Social workers (n=13) | Qualitative | Individual and focus group interviews | n/a | Differing perceptions of palliative care emerged. Negative associations of palliative care were identified. Very few clients with PD are referred to specialist palliative care specifically for management of their symptoms, which must prevent holistic care. Social workers have an important yet underdeveloped role in identifying and addressing palliative care needs. |
| **Hasson, F. et al. (2010)** | To explore former carers’ lived experiences of palliative and end-of-life care. | Family caregivers (n=15) of patients who had died with Parkinson’s disease. | Qualitative | Individual interviews | n/a | Lack of communication, knowledge and coordination of services resulted in many people caring for someone with PD not accessing specialist palliative care services. Participant’s also reflected on the physical and psychological impact of caring in the advanced stage of PD. |
| **Waldron, M. et al. (2010)** | To explores the views of allied health professionals in delivering rehabilitation in palliative  care to people with Parkinson’s disease. | Allied health professional (n=12), from both generalist and specialist settings | Qualitative | Focus group interviews | n/a | Participants viewed palliative care as holistic care, however, the rehabilitation care they provided was impeded by a number of personal and organisational barriers, and negative perceptions. There is a need to educate professionals in the principles of palliative rehabilitation, combined with providing targeted resources to promote shared care and responsibility. |
| **THEME: SPECIFIC GROUPS (Same sex couples)** | | | | | | |
| **Glacken et al. (2008)** | To explore the grief experience of same sex couples. | Individuals (n=7) whose partners died from an AIDS/HIV-related illness. | Qualitative | Individual interviews | n/a | Five themes emerged that captured the bereavement experience: tacit acknowledgement; sculpting the distress; multiple losses; seeking support; and journeying anew. Many of the participants experienced disenfranchised grief. Health care professionals need to consider their approach to people who identified themselves as gay or lesbian, if they are to provide support structures (formal and informal) to meet their unique needs. |
| **THEME: SPECIFIC GROUPS (Travellers)** | | | | | | |
| **McQuillan, R. et al. (2007)** | To explore the indigenous ethnic minority group, Irish Travellers and palliative care services in Ireland. | Specialist palliative care service providers; Travellers (5 groups, n=?) | Mixed methods | Questionnaire (n=81); individual and focus group interviews (n=16) | n/a | Common themes emerged- low use of palliative care services by Travellers, concerns of both Travellers and specialist palliative care staff about Travellers education and literacy ability to deal with health care services, the role of the family, and demonstrative expressions of grief by Travellers. Both sides were aware of their lack of information about the other. |
| **THEME: SPIRITUALITY** | | | | | | |
| **Bailey, M.E. et al. (2009)** | To describe nurses’ experiences of delivering spiritual support in a palliative care setting. | Specialist palliative care nurses (n=22) | Qualitative | Individual interviews | n/a | Five sub-themes: understanding spirituality; the art of nursing in spiritual care; education and learning; the challenges of spiritual caring; and the dimensions of time. The challenges of assessing spiritual needs were also reported. Participants described the creation of a spiritual tapestry that ‘weaves’ together care and compassion with skills & knowledge in their nursing practice. |
| **Kernohan, G. et al. (2007)** | To assess patients’ spiritual needs and perceptions of a chaplaincy service. | Patients (n=62) | Mixed methods | Case review; Questionnaire |  | Findings suggest that the Standards for Hospice and Palliative Care Chaplaincy (2003) were useful for assessing and addressing spiritual needs. Participants, of whom 92% had a faith in God or a Higher Being, highlighted their top six spiritual needs as: to have time to think; to have hope; to deal with unresolved issues; to prepare for death; to express true feelings without being judged; and to speak of important relationships. Majority of participants felt that their spiritual needs had been met. |
| **MacConville, U. (2006)** | To "map" understandings of religion and spirituality in an Irish palliative care setting |  | Qualitative | a cartographic approach | n/a | Aspects of religion and spirituality have been explored within a multi-layered Irish cultural setting to reveal a complex landscape--a landscape that is changing but which draws upon the past in shaping the present. |
| **THEME: SYMPTOMS (CACHEXIA)** | | | | | | |
| **Reid, J. et al. (2009)** | To investigate the tensions over food that exists between patients with advanced cancer with cachexia and their families. | Patients with advanced cancer living with cachexia (n=8); family members (n=8) | Qualitative (Heideggerian phenomenologic approach) | Individual interviews | n/a | Findings highlight the anxiety that surrounds eating and the distress it causes to patients and their families. This strain can escalate into arguments over food, causing negative repercussions for patients and their family members. |
| **Reid, J. et al. (2009)** | To investigate the perceptions of patients and family members with regard to care received for cancer cachexia. | Patients (n=15) with advanced cancer who had primary cachexia & family members (n=12) | Qualitative | Interviews | n/a | A major finding was ‘lack of response from health care professionals’ in relation to cancer cachexia management. Participants reported wanting three things from healthcare professionals: profound weight loss acknowledged; information about it and why it was happening; and interventions to deal with it. |
| **THEME: SYMPTOMS (DELIRIUM)** | | | | | | |
| **Meagher, D. et al. (2012)** | To examine features that characterise subsyndromal delirium and persistent delirium over time | Adults with DSM-IV delirium (n=100) | Quantitative | Assessments | Delirium Rating Scale- Revised-98 (DRS-R98) and Cognitive Test for Delirium (CTD) | Full syndromal delirium was significantly more severe than subsyndromal delirium for DRS-R98 thought process abnormalities, delusions, hallucinations, agitation, retardation, orientation, attention, and short- and long-term memory items, and CTD attention, vigilance, orientation and memory. Persistent full syndromal delirium had greater disturbance of DRS-R98 thought process abnormalities, delusions, agitation, orientation, attention, and short- and long-term memory items, and CTD attention, vigilance and orientation. |
| **Leonard, M. et al. (2011)** | To study phenomenological and neuropsychological profile across motor variants of delirium in a palliative care unit. | Patients meetings DSM-IV criteria for delirium (n=100) | Quantitative | Standardised assessments | DRS-R-98; Cognitive Test for Delirium (CTD); Delirium Motor Checklist (DMC); Delirium Motor Subtype Scale (DMSS); Ease of Ward management Scale (EOWM); Delirium Etiology Rating Checklist | Motor variants in delirium have similar cognitive profiles, but mixed cases differ in expression of several non-cognitive features. Similar to previous findings, patients with both hyperactive and mixed subtypes received greater attention and antipsychotic medication than their hypoactive counterparts. |
| **Godfrey, A. et al. (2010)** | To determine the use and feasibility of accelerometry-based monitoring and to examine a discrete multi-resolution signal analysis technique to determine motoric subtypes in patients with DSM-IV delirium. | Patients (n=34) | Quantitative | Assessments | (24-h accelerometer-based monitoring) | Of the 34 patients included, 25 met criteria for DSM-IV delirium while 9 were non-delirious comparison subjects with equivalent medical diagnoses receiving treatment in the same setting. It was concluded that accelerometry-based measurement of a delirious cohort within a palliative setting is both a reliable and feasible method of continuous monitoring. Of the activities performed by the patients, periods of standing proved to be the most discriminatory in determining between each subtype. |
| **Meagher, D. et al. (2010)** | To explore the impact of an educational workshop upon attitudes towards pharmacotherapy for delirium. | Health personnel (n=66) | Quantitative | Questionnaire | n/a | Most respondents reported psychotropic use with variable frequency. Antipsychotic use was inversely related to perception of supporting evidence. Post-workshop concerns regarding extrapyramidal effects were reduced with a more positive general attitude towards pharmacological interventions. |
| **Meagher, D. et al. (2010)** | A comparison of neuropsychiatric and cognitive profiles in delirium, dementia, comorbid delirium-dementia and cognitively intact controls. | Adults (n=?) with DSM-IV delirium, dementia, comorbid delirium-dementia and cognitively intact controls | Quantitative | Assessments | Revised Delirium Rating Scale (DRS-R98) and Cognitive Test for Delirium (CTD) | Delirium and comorbid delirium-dementia groups had comparable DRS-R98 and CTD total scores, which were greater than in dementia or control groups. On the DRS-R98, multiple non-cognitive symptoms, inattention and disorientation were more severe in delirium groups compared with dementia-alone. Patients with dementia differed from both delirium groups on the CTD test of attention. Spatial span backwards was significantly lower in all patients with cognitive impairment (delirium, comorbid delirium-dementia, dementia alone) compared to controls, whereas spatial span forwards distinguished delirium groups from dementia. |
| **Leonard, M. et al. (2009)** | To assess and compare mood states as they relate to onset of delirium. | Patients (n=100) | Quantitative | Assessments | DSM–IV criteria; HADS; Confusion Assessment Method (CAM); Memorial Delirium Assessment Scale (MDAS); Mini-Mental State Exam (MMSE) | Overall, 51% experienced either major depression or delirium. Most patients with syndromal delirium also met criteria for major depressive illness, and 50% of those with depression had delirium or subsyndromal delirium (SSD). Delirium symptoms were less common in patients with major depression than depressive symptoms in patients with delirium or SSD. |
| **Ryan, K. et al. (2009)** | To determine the sensitivity and specificity of the Confusion Assessment Method (CAM) in diagnosing delirium when used by Non-Consultant Hospital Doctors (NCHDs) working in a specialist palliative care unit. | Pilot study: patients (n=32); main study: patients (n=52) | Quantitative | Assessments | CAM; Revised Delirium Rating Scale (DRS-R-98); Cognitive Test for Delirium (CTD); Memorial Delirium Assessment Scale (MDAS) | Results suggest that the CAM is a valid screening tool for delirium in the palliative care setting but its performance is dependent on the skill of the operator. NCHDs require a certain standard of training before becoming proficient in its use. |
| **Leonard, M. et al. (2008)** | To evaluate factors related to reversibility and mortality in consecutive cases of Diagnostic and Statistical manual of Mental Disorders (4^th^ Ed) delirium occurring in palliative care patients. | Patients with delirium (n=121) | Quantitative | Assessments | Delirium Rating Scale-Revised-98 (DRS-R-98); Cognitive Test for Delirium (CTD); EOWM | Findings suggest that reversible delirium is distinguishable by young patient age, less severe cognitive disturbance and absence of organ failure as a cause of delirium. Both the DRS-98 and CTD total scores were worse in the irreversible group. This study along with others identified clinical features that can allow greater precision in prospectively identifying less reversible or ‘terminal’ delirium. |
| **Leonard, M. et al. (2008)** | To provide an expert review of delirium in the context of palliative care | n/a | Systematic review | Review | n/a | Delirium occurs commonly in the context of palliative care where it is likely to cause heightened distress for patients, carers, and families alike, and make interpretation of pain and other symptoms extremely difficult. There is a profound dearth of rigorous studies on delirium in this setting. Ambiguous terminology, varying definitions in internationally recognized classification systems, and failure to use validated assessment tools found. |
| **Meagher, D. et al. (2008)** | To validate a new approach to motor subtyping in delirium based on data from a controlled comparison of items from three | Cancer patients (n=50) & non-delirious comparison subjects (n=52) | Quantitative | Assessments; questionnaires | Delirium Rating Scale-Revised-98; Cognitive Test for Delirium; Delirium Motor Checklist | The new motor subtyping schema which was derived from existing schema for delirium is relatively simple and based on a checklist that can be rated by nursing staff. Findings suggest that the new scale relates closely to the prevailing concept of delirium than other measures such as DRS-R98 and the Cognitive Test for Delirium. |
| **Meagher, D. et al. (2008)** | To evaluate delirium phenomena | Palliative care group | Quantitative | Assessments | 30-item Delirium Motor Checklist (DMC) | In delirium, motor disturbance was present in 100% by DMC versus 92% by DRS–R98 motor items; the DMC motor items also significantly distinguished delirium from control subjects. Motor subtype classification (hyperactive, hypoactive, mixed, and none) varied among the four methods, with low concordance across all four methods and 76% concordance for pairwise comparisons. The DRS–R-98 identified the most hypoactive delirium cases. |
| **Leonard, M. et al. (2007)** | To (a) test the applicability of accelerometry in highly morbid patients with delirium; (b) test the correlation of accelerometer readings with observed gross movement; (c) compare quantitative and qualitative motion in motorically defined groups. | Patients (n=3) | Quantitative | Assessments | DRS-R98; Memorial Assessment Schedule; | The procedures were well tolerated and motor presentations were readily distinguished using the accelerometer-based measurements. The system was capable of identifying static versus dynamic activity and the frequency of changes in posture. Electronic motion analysis concurs with observed gross movement and can distinguish motorically defined subtypes according to quantitative and qualitative aspects of movement. |
| **Meagher, D. et al. (2007)** | To investigate the relationship between cognitive and non-cognitive delirium symptoms and test the primacy of inattention in delirium. | Individuals (n=100) with delirium | Quantitative | Assessments | Delirium Rating Scale–Revised–98 (DRS–R98) and Cognitive Test for Delirium (CTD | Sleep–wake cycle abnormalities and inattention were most frequent, while disorientation was the least frequent cognitive deficit. Patients with psychosis had either perceptual disturbances or delusions but not both. Neither delusions nor hallucinations were associated with cognitive impairments. Inattention was associated with severity of other cognitive disturbances but not with non-cognitive items. CTD comprehension correlated most closely with non-cognitive features of delirium. |
| **THEME: SYMPTOMS (NUTRITION)** | | | | | | |
| **Gray, R.T. et al. (2011)** | To assess the relationship between nutritional factors and 30-day mortality in patients undergoing SEMS insertion for palliation of oesophageal cancer. | Patients (n=53) | Qualitative | Retrospective clinical case note review | n/a | Fifty-six stents were inserted into 53 patients. Thirty (56.6%) patients tolerated an oral diet enhanced with supplement drinks whereas 43.4% patients required more invasive forms of enteral and parenteral support. BMI, calorific intake and swallowing capacity were not predictors of survival. Invasive nutritional support itself was not predictive of 30-day mortality (P=0.74). |
| **Watson, M. et al. (2010)** | To compare attitudes of hospice staff towards weight loss and weight assessment in the hospice setting with those of patients with advanced malignancy in the hospital outpatient setting. | Hospices (n=71 doctors, 74 nurses); Oncology outpatients (n=129) | Quantitative | Questionnaire | n/a | Weighting practices vary across hospices in UK (NI) and Ireland and patients attending the majority of hospices are hardily weighed. While there is reluctance on the part of many hospice staff to weigh patients, most patients with advanced malignancy in the hospital setting do not report weight measurement to be upsetting. |
| **THEME: SYMPTOMS (PAIN)** | | | | | | |
| **Barry, H. et al. (2012)** | To explore the knowledge, attitudes and beliefs that nursing home managers hold with regard to the assessment and management of pain in residents with dementia and to determine how these may be affected by the demographic characteristics of the respondents. | Nursing home managers (n=95) | Quantitative | Questionnaire | n/a | Nearly all respondents (96%) provided care to residents with dementia, yet only 60% of managers claimed to use pain treatment guidelines within their nursing home. Nursing home managers were uncertain about how to manage pain in residents with dementia, demonstrating similar concerns about the use of opioid analgesics to those reported in previous studies about pain in older people. Managers who had received recent training were less likely to have concerns about the use of opioid analgesia than those who had not received training. |
| **Rafferty, M.N. et al. (2012)** | To assess the economic cost of chronic pain in Ireland. | Individuals with chronic pain (n=140) | Quantitative | Assessments | Chronic Pain Grade Questionnaire  (CPG); Client Services Receipt Inventory  (CSRI) | Mean cost per chronic pain patient calculated per year across all grades of pain, with mean costs increasing according to the severity of pain. Those with clinically elevated depression scores had costs that were twice as high as people who scored below the depression cut-off score. Chronic pain services in Ireland are generally under resourced. Improved coordination and better management of patients via interdisciplinary pain rehabilitation program is essential. |
| **Alaouabda, N. et al. (2011)** | To describe the pattern of chronic pain practice (CPP) among consultant anaesthetists in Ireland. | Consultant anaesthetists (n=127) | Quantitative | Questionnaire | n/a | While 28% of responding anaesthetists were involved in CPP, in the majority of cases, this accounted for less than 20% of their clinical time. 39% of those involved in CPP had previous training in chronic pain management. The types of CPP included nerve blocks (67%) and pharmacological treatment (44%) in non-cancer pain (67%) and cancer pain (61%) patients. Epidural steroid injection was the most commonly practiced intervention (89%). |
| **Brown et al. (2011)** | To develop the nursing practice context to enable more effective pain management with older people. | Nursing staff (n=48) | Emancipatory Action Research | Focus groups; reflective sessions; workshops | n/a | 3 key themes (psychological safety, leadership, and oppression) and 4 subthemes (power, horizontal violence, distorted perceptions, and autonomy) were found to influence the way in which effective nursing practice was realised. Within the theme of ‘context’, effective leadership and the creation of a psychologically safe environment were key elements in the enhancement of nursing practice. |
| **Cornally, N. et al. (2011)** | To explore the help-seeking behaviour, individual characteristics, attitudes, and beliefs of older adults with chronic pain in an Irish community setting. | Older adults with chronic pain (n=72) | Quantitative | Questionnaire | Level of Expressed Need Questionnaire; Pain Attitudes Questionnaire; Pain Beliefs Questionnaire | Individual characteristics associated with help-seeking behaviour were female gender, increasing age, higher education, living alone, and severe pain. High levels of stoicism indicated that participants were more likely to believe they had superior pain control and courage in the face of pain and were not willing to disclose their pain to others. Participants had moderate age-related beliefs about the origin of pain. |
| **Rowley D. et al. (2011)** | To identify this cohort of patients within our practice, to review the use of opioid analgesia in these patients, to identify the characteristics of this patient group, and to review the literature on the topic. | Patients (n=12) referred to the palliative care service with cancer pain who were on MMT. | Qualitative | Retrospective clinical case note review | n/a | This study demonstrated that significant difficulties were experienced in achieving pain control in this patient group. Although half of the patients were not on opioid analgesia on referral to palliative care, all required opioids to achieve pain control. Furthermore, multiple analgesic agents were required in 70% of patients. |
| **Duignan, M. et al. (2009)** | To compare how barriers to pain management in emergency departments are perceived by nurses in the Republic of Ireland and in the United States. | Hospital sites (n=5); Emergency nurses in the HSE area (n=81) | Quantitative | Questionnaire | n/a | Most nurses in the study (n=67, 83 per cent) had undertaken no pain management training. Most barriers to pain management identified by the participants can be described as ‘organisational’. The most frequently identified barrier in this study was the inability to give patients analgesia until medical diagnosis was made. Lack of time to assess and control pain adequately was identified by RoI nurses as the second largest barrier. Other challenges reported. |
| **THEME: SYMPTOMS (PHYSICAL)** | | | | | | |
| **White, C. et al. (2009)** | To determine which symptoms experienced by patients admitted to a specialist palliative care unit are self-reported (SR) and which are only detected with systematic questioning (SQ). | Patients’ charts (n=50) | Qualitative | Retrospective clinical case note review | n/a | The most common SR symptoms were pain, bowel disturbance, nausea or vomiting, mobility problems & loss of appetite. The most common SQ symptoms were weight loss, fatigue, loss of appetite, mobility problems, edema/ lymphedema, oral symptoms, confusion/ memory loss, sleep problems, bowel disturbance, drowsiness, and low mood. |
| **THEME: SYMPTOMS (PSYCHOLOGICAL)** | | | | | | |
| **Anderson, T. et al. (2008)** | To assess whether the use of CBT techniques in hospice patients is an acceptable intervention. | Patients (n=11); in-patients & day hospice | Mixed methods | Assessment; semi-structured interviews | Hospital Anxiety and Depression Scale (HADS) | This study showed that a palliative care professional with short training in CBT was usefully able to apply CBT techniques to hospice patients with mild-to-moderate anxiety or depression. |
| **Sharp, L. et al. (2012)** | To investigate associations between cancer-related financial stress and strain and psychological well-being | Individuals post-diagnosis with breast, prostate & lung cancer (n=654) | Quantitative | Questionnaire | n/a | Cancer-related financial stress and strain were consistently associated with increased risk of adverse psychological outcomes (depression, anxiety and distress). |
